# Access to healthcare for Trans, Travestis, and Gender-Diverse people in Latin America: a scoping review

**DOI:** 10.3389/fpubh.2025.1710952

**Published:** 2026-01-19

**Authors:** Livia Maria de Souza Gonçalves, Norberto Rech Bonetti, Helena Moraes Cortes, Beverley D. Glass, Luciano Soares

**Affiliations:** 1 Postgraduate Program in Pharmaceutical Services and Policies (PPGASFAR), Department of Pharmaceutical Sciences, Federal University of Santa Catarina, Florianópolis, Brazil; 2 Professional Master's Program in Family Health (PROFSAÚDE), Department of Pharmaceutical Sciences, Federal University of Santa Catarina, Florianópolis, Brazil; 3 Professional Master's Program in Family Health (PROFSAÚDE), Postgraduate Program in Nursing (PEN), Universidade Federal de Santa Catarina, Florianópolis, Brazil; 4 College of Medicine and Dentistry, James Cook University, Townsville, QLD, Australia

**Keywords:** community health services, gender-affirming care, health inequities, health policy, health services accessibility, health services for transgender persons, transgender persons

## Abstract

**Background:**

Trans, Travestis, and Gender-Diverse (TTGD) populations in Latin America face substantial barriers to equitable healthcare. *Travesti* refers to a regionally specific gender identity with distinct cultural and political meanings.

**Objective:**

This scoping review aimed to map and characterize the literature and factors influencing access to health services and goods (such as medicines, diagnostic tests, and other resources for these populations).

**Methods:**

Following Joanna Briggs Institute (JBI) methodology and Preferred Reporting Items for Systematic Reviews and Meta-Analyses extension for Scoping Reviews (PRISMA-ScR) guidelines, a comprehensive search was conducted in SciELO, PubMed, LILACS, Web of Science, and Scopus. Paired screening and data charting were guided by a multidimensional access framework encompassing enabling and disabling factors, health needs, and individual behaviors. Inclusion criteria comprised primary studies on healthcare access for TTGD people in Latin America.

**Results:**

A total of 115 studies were included, all published between 2007 and 2024, although no publication date restrictions were applied. Disabling factors included undertrained professionals, pathologizing protocols (e.g., requiring diagnoses to access gender-affirming services), institutional transphobia (e.g., disrespect of social names and pronouns), limited funding for gender-affirming care, fragmented services, weak integration with primary care, inconsistent recognition of social names, and geographic or regional access gaps. Health needs were often unmet. Normative needs included gender-affirming procedures, hormone management, and comprehensive care. Felt needs encompassed humanized care, access to medicines and prostheses, chronic disease management, and psychological support. Expressed needs included emergency care and vaccination. Individual health behaviors reflected resilience and adaptation. Economic vulnerability and systemic exclusion (e.g., denial of care in public health facilities and cisnormative protocols) led many to avoid formal care and seek informal, peer-based, or self-managed alternatives. Access-enabling strategies included services involving TTGD professionals and inclusive policies, mostly implemented at municipal or institutional levels, promoting safe spaces, non-trans-pathologizing care, autonomy, and user collaboration.

**Conclusion:**

The findings highlight the importance of inclusive policies addressing cis-heteronormative norms and fostering community participation at all levels of healthcare, particularly in primary care. Key pathways to achieving equitable healthcare include structural reforms, investment in provider training, and the adoption of depathologized, rights-based models centered on TTGD leadership.

**Systematic review registration:**

osf.io/4zvse.

## 1 Introduction

Trans, Travestis, and Gender-Diverse (TTGD) people face persistent barriers to healthcare access (1–5). Beyond general health needs, TTGD populations often require gender-affirming services such as hormonization and body modification procedures (6). However, access to these services is often limited due to unmet health needs and experiences of discrimination (1–3, 5, 7, 8).

Trans, Travestis, and Gender-Diverse (TTGD) individuals have a gender identity that differs from the gender assigned at birth. This review adopts the term “TTGD people” as essential to a decolonial perspective, highlighting the political and cultural relevance of regional terminology and the plurality of identities, while acknowledging potential interpretive differences for international audiences. “Trans” is an umbrella term encompassing multiple gender identities. Although “Travesti” may carry pejorative connotations in certain contexts, in Latin America, it designates a specific transfeminine identity with distinct cultural and political significance. The term “Gender Diverse” refers to individuals whose gender identities or expressions diverge from binary gender norms (9, 10). In this regional context, “hormonization” denotes the use of hormones to affirm gender diversity as a human right and to contest the biomedical pathologization of Trans bodies (11).

Latin America presents a unique context for understanding healthcare disparities among TTGD populations. Although several countries have recognized health as a universal right and adopted policies to support TTGD healthcare, such as Brazil's National Policy for Comprehensive LGBT Health (12), Argentina's Gender Identity Law (13), and Uruguay's Ley Integral para Personas Trans (14), structural challenges persist, including fragmented health systems, underfunding, regional disparities (15, 16), and high levels of violence against TTGD people. The region accounts for over 70% of reported murders of Trans individuals worldwide (17). TTGD people also frequently face institutional discrimination in healthcare settings, including misgendering, disrespect, and denial of care (2, 18–20).

Previous reviews have examined aspects of TTGD healthcare, but important limitations remain. Many focus solely on Brazil, specific levels of care, or primarily on lesbian, gay, and bisexual populations, with limited attention to trans experiences (2, 5, 21–23). Even in global reviews including Latin America, the region is often subsumed within broader international contexts, limiting depth and specificity (3, 4, 23, 24). Consequently, comprehensive knowledge on how access to healthcare for TTGD people is framed, discussed, and studied across Latin America remains limited.

A scoping review is particularly suited to map these patterns and gaps, identify key concepts and types of evidence, and provide a comprehensive overview of this complex, underexplored area (25).

This study's framework was guided by Andersen's Behavioral Model of Health Services Use due to its multidimensional perspective on healthcare access, encompassing both individual and contextual factors (26), and is recognized as the most important and frequently cited theoretical model in this field of research (27). We adapted selected components, particularly enabling factors, with the dimensions of information, acceptability, and adequacy proposed by Soares (28) and Sanchez and Ciconelli (29), alongside an expanded concept of health needs based on Bradshaw (30), to provide a comprehensive, context-sensitive lens for examining health access among TTGD populations in Latin America. By mapping populations, countries, and factors influencing healthcare access, this review provides an overview of the current state of knowledge, highlights research gaps, and informs context-sensitive approaches to healthcare provision and policy discussions grounded in the realities of TTGD communities in the region. Anticipated limitations include language restrictions, potential publication bias, and heterogeneity across included studies, which may affect the completeness and comparability of the evidence.

## 2 Methods

We conducted a scoping review using a systematic and structured approach to comprehensively map the existing literature, characterize factors related to, and identify knowledge gaps regarding access to healthcare services and goods for TTGD people in Latin America. The guiding question for the review was “What are the characteristics of the scientific literature and the factors related to access to health services and goods for TTGD people in Latin America?” The Population, Concept, and Context framework guided the review, defining Trans, Travestis, and Gender-Diverse people as the Population, healthcare access as the Concept, and Latin American health services as the Context.

The methodology followed the Joanna Briggs Institute (JBI) Manual for Scoping Reviews guidelines (25) and adhered to the Preferred Reporting Items for Systematic Reviews and Meta-Analyses extension for Scoping Reviews (PRISMA-ScR) checklist (31), ensuring a rigorous and transparent process across all stages of the review. The research protocol was developed and prospectively registered on the Open Science Framework (osf.io/4zvse) before conducting the review, with updates made as necessary (32).

### 2.1 Search strategy

A comprehensive search was conducted between May 5 and 9, 2024, across the SciELO, PubMed, LILACS, Web of Science, and Scopus databases. Controlled vocabularies (DeCS/MeSH) and relevant keywords, including synonyms, were used to maximize the retrieval of studies focused on Latin America. Searches were carried out in English, Portuguese, and Spanish, with no date restrictions. The process followed the JBI three-step strategy: initial identification of terms, expanded database searches, and screening of included studies' reference lists. Full search strategies are available in Supplementary material.

The strategy was designed to map the literature on access to health goods and services for TTGD people living in Latin America. Boolean operators and tailored strings were adapted to each database. In LILACS and SciELO, DeCS terms and commonly used keywords were combined in strings written in Portuguese, Spanish, and English. In PubMed, MeSH terms and common English keywords were used. For Web of Science and Scopus, English strings included synonyms and entry terms from MeSH. In databases not specifically focused on Latin American research, geographic delimiters such as regional MeSH terms or the explicit names of Latin American countries were included to refine the scope. The search strategy applied no date filters.

### 2.2 Study selection

Duplicates were removed using EndNote software and manual checks. Prior to screening, a calibration exercise was performed to ensure at least 75% agreement between reviewers. Subsequently, two reviewers independently screened titles, abstracts, and full texts. Any discrepancies were resolved through consensus or by consulting a third reviewer. Inclusion criteria were:

(a) studies addressing access to healthcare goods and services for TTGD or Lesbian, Gay, Bisexual, Trans, Queer, Intersex, Asexual, Pansexual, Non-binary, and other (LGBTQIAPN+) populations, with findings specifically reported for TTGD participants; thus, studies in which TTGD data could not be separated from broader LGBTQIAPN+ data were not included;
(b) primary research, including empirical qualitative, quantitative, and mixed-methods studies, excluding reviews, commentaries, editorials, or opinion pieces;
(c) availability of abstracts to allow screening; and
(d) participants residing in Latin American countries.

Gray literature and policy reports were not included, as they did not meet criterion (b); as the review aimed to analyze peer-reviewed scientific evidence. These criteria ensured a focus on original evidence addressing healthcare access for TTGD people in the region and supported the feasibility of the screening process, in line with JBI guidance (25).

**Figure 1:**
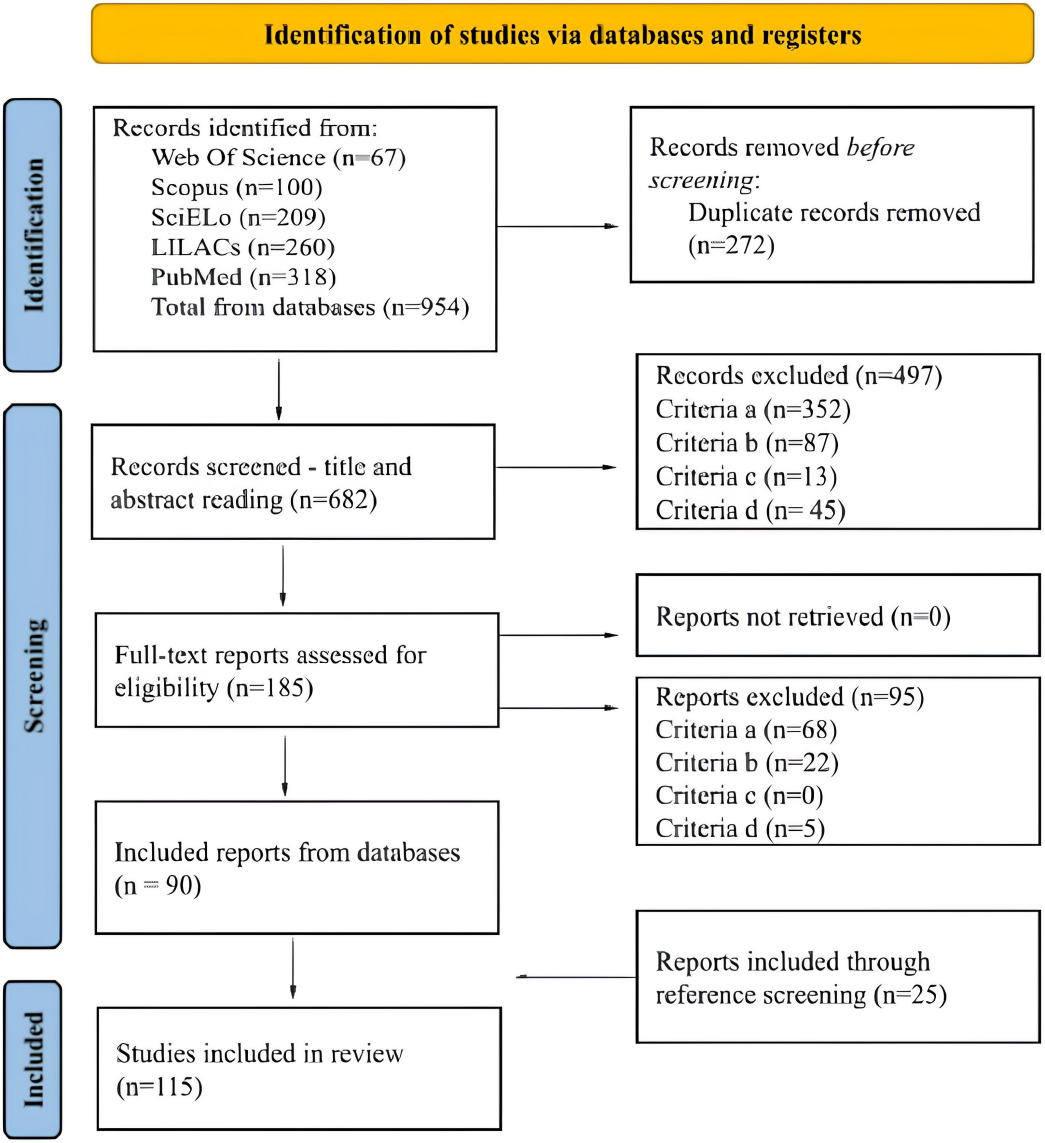
PRISMA-ScR flow diagram illustrating the study selection process.

The search yielded 954 records, of which 272 duplicates were removed. A total of 682 titles and abstracts were screened, and 185 full-text articles were assessed for eligibility. Of these, 497 were excluded during title and abstract screening and 95 during full-text review based on the eligibility criteria. Most exclusions occurred because TTGD-related results could not be distinguished from those of the broader LGBTQIAPN+ population (criterion a) or because the records did not constitute primary research (criterion b). A total of 90 studies were included through database searches and an additional 25 through reference screening, resulting in 115 studies. Among the excluded records, eighteen retrieved through the search and published between 1994 and 2006 were also removed during screening for not meeting the eligibility criteria, mostly due to criterion a. This helps explain why all included studies were published from 2007 onward. The full flow of identification, screening, and inclusion is presented in the PRISMA-ScR diagram (Figure 1).

### 2.3 Data charting and analysis

Data charting was conducted independently by pairs of reviewers using a standardized Excel^®^ sheet, with consistency ensured through pilot testing (25). Extracted data included study characteristics, population, methodology, and dimensions based on the conceptual framework integrating Andersen's Behavioral Model (26) with further health access domains (28, 29). Information on the first author's field of graduation was obtained by consulting scientific platforms (ORCID, ResearchGate, and CNPq Lattes Curriculum) and official institutional websites, and authors were contacted when additional information was required.

Data charting was conducted using three tools: a general data form for all studies and specific forms for qualitative and quantitative data. Mixed-methods studies were extracted using both forms, ensuring that all relevant data were captured appropriately.

A general data charting form was developed to capture essential descriptive and contextual information from each included study. This form comprised variables related to study identification (e.g., title, authors, year, journal, and language), methodological features (e.g., study design, type of research, and population), and contextual characteristics (e.g., authors' institutional affiliation, country, and funding source). It also included fields to specify whether the study addressed health goods and services for Trans people exclusively or for both Trans and cis populations, as well as the level of healthcare involved. Finally, the form recorded details on population subgroups, such as transfeminine, transmasculine, and gender-diverse participants, ensuring comprehensive mapping of the study's scope and context.

Data charting templates and examples are provided in Tables 1, 2 (qualitative data). The quantitative extraction form retained the same core categories as the qualitative template, excluding qualitative methodological details. In addition, it included variables specific to quantitative studies (Table 3).

**Table 1:** Data charting template for qualitative studies.

**Authors**	**Year**	**Enabling factors**								
		**Availability/ Accessibility**		**Payment Capacity/ Financing**		**Information**		**Acceptability**		**Adequacy**
		**Users**	**HCP**	**Users**	**HCP**	**Users**	**HCP**	**Users**	**HCP**	
Engelman M.	2007	Sex workers face difficulty accessing services during the day; assigned to men's wards; social names not respected. Health exclusion and self-exclusion related to stigmatization and discrimination are present in Mental Health Services	ND	Participants can often afford healthcare, but structural vulnerabilities, labor exclusion, and gender-based discrimination limit access to private coverage or funding for body-related care	ND	ND	ND	Experience stigma, rejection, and poor treatment in health services; some report being unfairly associated with HIV due to assumptions about Trans identity	ND	ND
Arán M, Zaidhaft S, Murta D.	2008	Reported delays and scheduling difficulties for gender-affirming surgeries due to institutional barriers	ND	ND	ND	ND	ND	Users generally accepted the outcomes of gender-affirming surgery and felt satisfied with the results, despite experiencing institutional barriers, occasional complications, or the need for additional care	ND	ND

HCP, Healthcare Professionals; ND, Not described.

**Table 2:** Extended data charting template for qualitative studies.

**Health needs**				**Individual health behaviors**		**Results summary**	**Qualitative methodological details**
**Normative**	**Felt**	**Expressed**	**Comparative**	**Users**	**HCP**		
ND	ND	ND	ND	Use of medications without endocrinological supervision, application of industrial silicone, and avoidance of health services	Authoritarian care with biomedical logic, without qualified listening. Lack of qualifications to treat travestis. Discrimination, stigma, and violence when treating users due to their gender identity, living with HIV, or being a sex worker	Participants reported barriers to accessing healthcare and reduced quality of care, including stigma and discrimination linked to gender identity and HIV status. Misconceptions among professionals contributed to exclusionary practices, leading to both institutional and self-exclusion from services	ND
Gender-affirming surgeries	Some participants identified gender-affirming surgery as a need, although this was strongly influenced by healthcare providers and did not reflect the priorities of all users	ND	ND	ND	Pathologization of Trans identities (psychiatric care for the diagnosis of transsexualism as a prerequisite for surgery)	Participants' experiences highlight how social exclusion and non-acceptance of gender diversity, rather than individual pathology, create distress. Challenges reflect broader limitations of rigid sex and gender classification systems and their exclusionary impact on Trans and gender-diverse people	ND

HCP, Healthcare Professionals; ND, Not described.

**Table 3:** Specific categories of data charting template for quantitative studies.

**Authors**	**Year**	**Procedures**	**Method details**	**Intervention**	**Duration of intervention**	**Analysis categories (outcomes)**
Galea, J. T., Kinsler, J. J., Salazar, et al.	2011	Observational Analytical	Cross-sectional	NA	NA	PrEP acceptability
Bittencourt, D., Fonseca, V., Segundo, M.	2014	Observational Descriptive	Cross-sectional	NA	NA	Search for health services and perception of quality of care

NA, Not Applicable.

The conceptual framework also underpinned the analysis. A detailed rationale for the theoretical foundation is provided in Supplementary material. Data were summarized using a basic deductive content analysis, consistent with JBI guidance (25). Extracted findings were independently categorized by each researcher according to the predefined framework rationale, with continuous comparison and without reinterpretation. This approach enabled a structured mapping of the evidence while respecting the methodological diversity and heterogeneity of the included studies.

Frequency counts were used to quantify the occurrence of publications by country, research methods, target populations, and other key elements within the literature. The geographic distribution of included studies was visually represented using Datawrapper (33).

Results are presented in tables and figures detailing study characteristics and dimensions related to healthcare access. Additionally, narrative summaries were provided within predefined sections aligned with the conceptual framework.

## 3 Results

A total of 954 records were identified across all databases searched, resulting in 115 studies included in the review. Information on records found in each database, duplicate removal, and the screening process is presented in the PRISMA-ScR flow diagram (Figure 1).

### 3.1 Study characteristics and findings

The 115 primary articles addressing health access for TTGD people in Latin America were published between 2007 and 2024.

**Figure 2:**
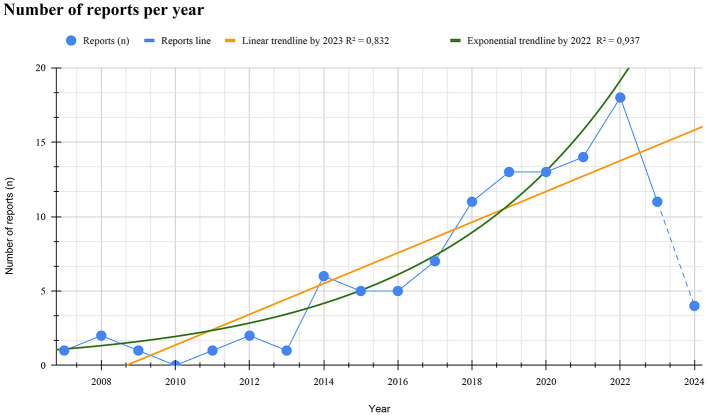
Number of reports per year. Annual distribution of reports (n) addressing access to health services and/or health products by TTGD populations in Latin America, from 2007 to 2024. Blue dots represent the number of reports per year, connected by a solid blue line until 2023 and a dashed line for 2024, reflecting incomplete data not included in the trendline calculations. The yellow line shows the linear trendline up to 2023 (*R*^2^ = 0.832), and the green line indicates the exponential trendline up to 2022 (*R*^2^ = 0.937).

There is a clear upward trend in publications beginning around 2013, with some yearly fluctuations and a peak of 18 studies in 2022 (Figure 2). The linear trendline indicates a strong positive correlation (*R*^2^ = 0.832), projecting continuous growth through 2023. However, there was a notable decline in the number of reports in 2023. Data for 2024 was incomplete since the search was limited to May 2024; therefore, excluded from the trendline analysis. The exponential trendline, calculated up to 2022, shows the strongest fit (*R*^2^ = 0.937) and highlights that the publication rate followed an exponential growth pattern until that year. After 2022, the trajectory shifted to a linear trendline, which provides a more accurate representation of the overall pattern through 2023.

**Figure 3:**
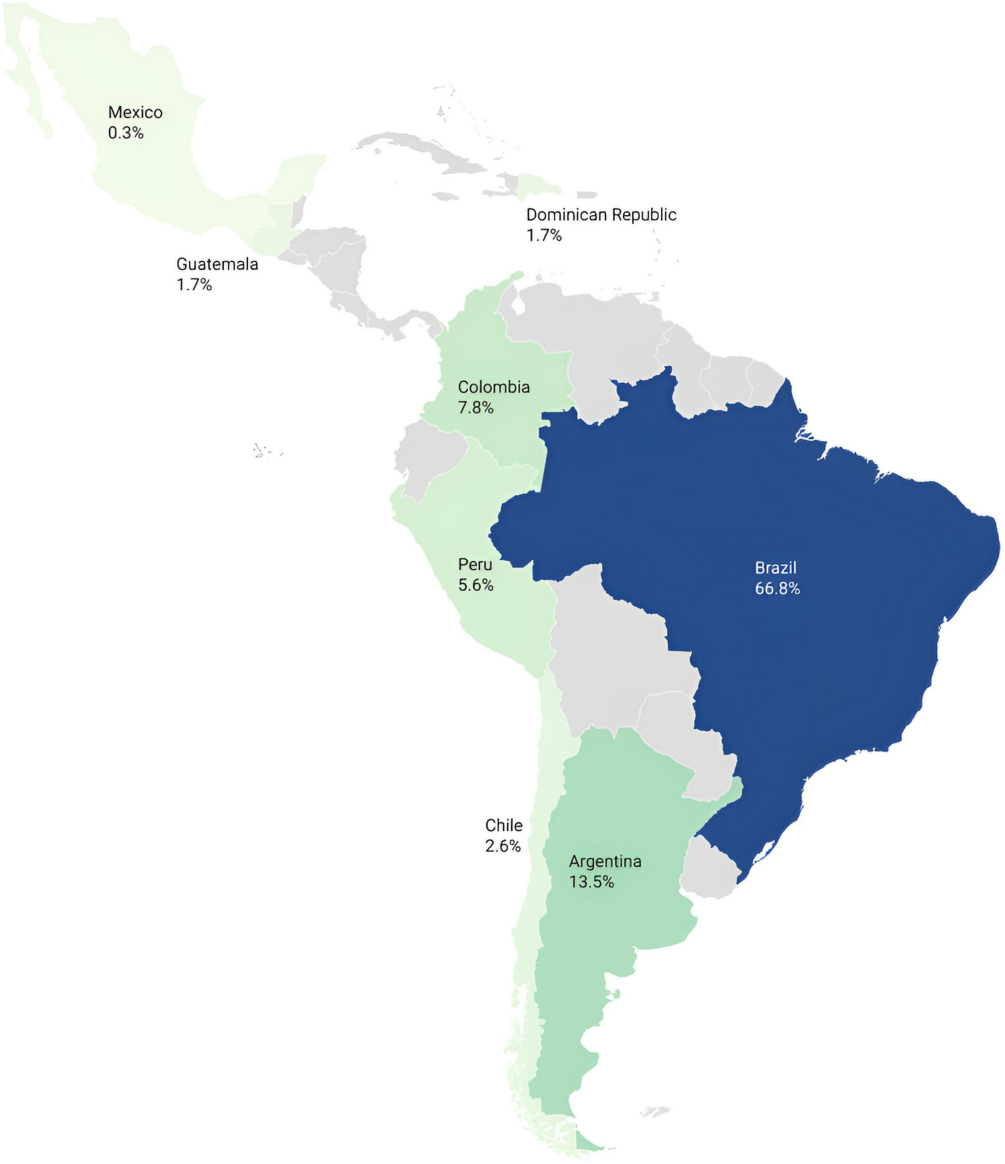
Geographic distribution of included studies (n = 115) on access to health services and/or health products for TTGD populations in Latin America. Darker shades indicate a higher proportion of studies conducted in each country. No studies were identified in countries shaded in gray. Figure generated using Datawrapper (33).

The geographical distribution of included studies (Figure 3) illustrates a strong concentration of publications in Brazil and Argentina, which account for over 80% of all records. This suggests that research on Trans health access in Latin America remains uneven, with limited representation from Central America and smaller South American countries, highlighting a geographic bias that should be considered when interpreting the regional scope of the findings. A total of 64 research institutions were identified, located mainly in Argentina, Brazil, and Colombia, with studies widely dispersed among them. Fundación Huésped led with 6.9% of publications, followed by Universidade Federal da Bahia, Pontificia Universidad Javeriana, and Faculdade de Ciências Médicas da Santa Casa de São Paulo, each with 4.3%.

The objectives, countries, types, and findings of the studies are presented in Table 4. Each article has been assigned a reference number (column “N”), which will be used to refer to the respective study throughout the Section 3.

**Table 4:** Study characteristics and findings.

**N°; Authors, year; Country**	**Study method**	**Objective**	**Results summary**	**References**
1; Engelman, 2007; Argentina	Qualitative	To identify key factors that hinder or facilitate access to health services and dignified care for sex workers, within the context of HIV/AIDS and other STIs, focusing on CABA	Participants reported barriers to healthcare access and reduced care quality, including a widespread misconception among professionals linking mental health conditions to HIV/AIDS. This view often misinterprets Travestis' behaviors as inherent issues, reinforcing stigma and gender discrimination, and contributing to institutional and self-exclusion	(34)
2; Arán, Zaidhaft, Murta, 2008; Brazil	Qualitative	To explore how trans identities are understood in health practices and examine the diversity of trans narratives	Trans identities were associated with profound suffering, not only from gender incongruence but primarily due to social marginalization. The study highlights the inadequacy of current sex/gender classifications, which rely on exclusionary frameworks shaped by prevailing cultural norms	(35)
3; Müller, Knauth, 2008; Brazil	Qualitative	To analyze the perceptions, emotions, and challenges Travestis face in accessing health services and receiving equitable care	Ten central themes emerged: language, body, discrimination, hospitalization, healthcare services, medications, HIV/AIDS, prejudice, coping strategies, and symbolic/physical violence. Findings emphasized the urgent need for professional training to counter the marginalization of Travestis, which poses serious public health risks	(36)
4; Bones Rocha et al., 2009; Brazil	Qualitative	To describe how Travestis, transgender, and gender diverse individuals perceive the care received through Brazil's Public Health System (SUS)	Identified themes included homophobia, reception and humanization, healthcare access, and system improvement suggestions. Participants noted the lack of provider preparedness and emphasized the use of social names as a key inclusion measure. Despite public policies, SUS principles were seen as inconsistently upheld	(37)
5; Galea et al., 2011; Peru	Qualitative and Quantitative	To assess the acceptability of Pre-Exposure Prophylaxis (PrEP)	High acceptability was linked to perceived efficacy, low side effects, and affordability. Concerns included potential risk compensation, stigma, discrimination, and mistrust of healthcare providers	(38)
6; Boyce et al., 2012; Guatemala	Qualitative	To identify barriers to sexual health services among gay, bisexual, heterosexual men, and trans individuals in Guatemala City, to inform service improvement	Public clinics were most used for affordability and accessibility, though many participants experienced discrimination, confidentiality breaches, and distrust. Trans participants preferred inclusive environments. Key barriers included discrimination, HIV fear, cost, and lack of social support	(39)
7; Sampaio, Coelho, 2012; Brazil	Qualitative	To examine the gender affirmation process from the perspectives of four individuals	Surgical and hormonal procedures and civil name changes were seen as essential for well-being. Major barriers included long SUS waiting lists, pre-op requirements, high private clinic costs, and legal limitations on identity documentation	(40)
8; Santos, 2013; Brazil	Qualitative	To understand how the NIAT hormonalization program functioned, the experiences of trans participants, and the views of those wishing for its return	Participants described limited dialogue with healthcare teams regarding hormone dosing and the imposition of rigid biomedical models. These conflicts highlighted the need to recognize trans identities as lived experiences rather than narrowly defined by medical protocols. Access to hormones was often prioritized over surgery	(41)
9; Bittencourt, Fonseca, Segundo, 2014; Brazil	Qualitative and Quantitative	To explore LGBT individuals' perspectives on healthcare access, considering the impact of living in slums	Access difficulties were linked to confidentiality breaches, discrimination, and personal beliefs about care. Health professionals showed resistance to discussing sexual diversity and struggled with their own biases, perpetuating silence on the topic	(42)
10; Báez, 2014; Colombia	Qualitative	To understand transgender individuals' experiences with healthcare providers in Bogotá	Access barriers included provider shortages and stigmatizing attitudes toward trans identities. Some sought care for safe gender affirmation, while others turned to self-medication. Psychological support was valued but often inconsistent. Findings revealed a tense and contradictory relationship with health services	(43)
11; Sánchez, Casquero, Chávez, Liendo, 2014; Peru	Qualitative and Quantitative	To assess knowledge, usage, side effects, information sources, access, and adverse effects of hormone use among transfeminine individuals, along with sociodemographic data	Few participants received medical guidance—only 11.8% had prescriptions, and 5.9% reported pharmacist guidance. Most (65.9%) relied on friends. Pharmacies were the main access point. Common side effects included breast tenderness, weight gain (88.2%), and reduced libido/erection (58.8%). The most used hormone was intramuscular dihydroxyprogesterone with estradiol (47.1%)	(44)
12; Socías et al., 2014; Argentina	Quantitative	To identify individual, structural, and environmental factors linked to healthcare avoidance among trans women in Argentina	40.7% avoided care due to their gender identity. Associated factors included police violence, internalized stigma, discrimination by providers or patients, and residence in Buenos Aires. Health insurance reduced avoidance	(8)
13; Socías et al., 2014; Argentina	Quantitative	To examine factors influencing engagement with Argentina's Gender Identity Law among trans women	57.5% obtained new gender-congruent IDs. Prior healthcare discrimination and undergoing gender affirmation procedures were positively associated with this engagement	(45)
14; Souza, Signorelli, Coviello, Pereira, 2014; Brazil	Qualitative	To present the therapeutic itineraries of Travestis in Santa Maria, Rio Grande do Sul	Participants largely avoided formal health services, preferring Afro-Brazilian religious spaces, perceived as nonjudgmental and protective regarding body modifications and sexual orientation	(46)
15; Aguayo-Romero et al., 2015; Colombia	Qualitative and Quantitative	To analyze how personal and social factors affect gender affirmation and body modification among trans people in Bogotá	Peers on social media provided hormone and injection information and services, often with adverse effects. Procedures occurred outside formal care due to access barriers, stigma, community norms, and individual choices	(47)
16; Souza et al., 2015; Brazil	Qualitative	To examine how violence experienced by Travestis relates to their healthcare trajectories	Narratives revealed systemic violence—from families to institutions—enforced through rejection of bodily difference, including within health services	(48)
17; Petry, 2015; Brazil	Qualitative	To understand trans women's experiences with hormonalization and gender affirmation processes within SUS	Participants met SUS criteria and reported satisfaction with outcomes despite some complications. Many self-medicated before formal access. Diagnosis as a “trans person” was a prerequisite	(49)
18; Roger et al., 2015; Brazil	Qualitative	To analyze transgender people's access to primary health care considering social vulnerability	Discrimination and resistance to using social names limited access. Care was often stigmatizing, and providers were unprepared. Safety was reported only in a trans-specific outpatient clinic	(50)
19; Souza, Pereira, 2015; Brazil	Qualitative	To present health care experiences of Travestis in Santa Maria, RS	Travestis avoided institutional care, favoring Afro-religious houses for their inclusive, nonjudgmental support of body modifications and gender identity	(51)
20; Barrington et al., 2016; Guatemala	Qualitative	To identify factors shaping HIV diagnosis timing, care linkage, and retention	MSM and trans women were disproportionately affected. Fear of HIV's physical and social impacts delayed testing and care. Stigma and limited job opportunities hindered retention; NGO testing improved early diagnosis	(52)
21; Lima, Cruz, 2016; Brazil	Qualitative	To explore how testosterone use shapes trans men's identities and healthcare experiences	Public services were seen as unwelcoming, rigid, and protocol driven. Biomedical models dominated care, focusing on diagnosing and confirming trans identities through fixed surgical and hormonal paths	(53)
22; Neer, 2016; Argentina	Qualitative	To identify obstacles and facilitators in implementing the Gender Identity Law by gender-affirming health professionals in Buenos Aires and La Plata (2012–2015)	Professionals created local strategies to comply with the law amid bureaucratic and resource limitations, while also facing tensions with trans users	(54)
23; Rocon et al., 2016; Brazil	Qualitative	To examine barriers faced by trans people in Greater Vitória/ES when accessing SUS services	Key barriers included disrespect for social names, discrimination, and the diagnostic requirement for gender affirmation, which obscures heteronormativity's role in marginalization	(55)
24; Tagliamento, Paiva, 2016; Brazil	Qualitative	To analyze trans people's access to Brazil's public health system under new policies	Services applied gender stereotypes and stigmatizing models, especially where staff were untrained, perpetuating unsafe care outside the public system	(56)
25; Bustamante et al., 2017; Peru	Quantitative	To evaluate access and availability of HIV self-testing among MSM and trans women in Peru	Self-tests were accepted but often unavailable or costly. Only clinical-use kits—lacking user instructions—were sold in pharmacies, posing major barriers	(57)
26; Ferreira et al., 2017; Brazil	Qualitative	To explore Travestis' healthcare experiences within SUS in Teresina-PI	All participants reported discrimination. Services reflected broader societal marginalization, demanding stronger integration, professional training, and critical reflection on exclusionary dynamics	(58)
27; Budhwani et al., 2017; Dominican Republic	Quantitative	To profile transgender female sex workers in the Dominican Republic and assess HIV knowledge, stigma, and condom use	Low HIV knowledge and high stigma correlated with lower condom use, especially with regular partners. Over 73% reported difficulties accessing healthcare	(59)
28; Ortega et al., 2017; Argentina	Qualitative	To analyze waiting experiences for hormonization among trans people in Buenos Aires public hospitals	Waiting times and fragmented care systems hindered treatment. Class and gender asymmetries shaped these experiences, and provider attitudes often reinforced stigma despite legal depathologization	(60)
29; Pinto et al., 2017; Brazil	Qualitative	To assess transgender women's views on access, stigma, and treatment in gender affirmation	Medicalization dominated care, excluding patients from decisions. Although seen as natural by professionals, trans women accepted stigma to access needed services	(61)
30; Pinto et al., 2017; Brazil	Qualitative and Quantitative	To estimate industrial liquid silicone (ILS) use among Travestis and transgender women and related factors	ILS use was high, often initiated early, especially among Travestis with low education. Nearly half had undergone the procedure, often with health consequences	(62)
31; Sehnem et al., 2017; Brazil	Qualitative	To explore nurses' perspectives on primary care provided to Travestis	Primary care was not a viable entry point due to unprepared services. Care initiatives were rare, fragmented, and mostly driven by individual nurse efforts	(63)
32; Costa et al., 2018; Brazil	Quantitative	To address transgender and gender-diverse people's health needs and access barriers in Brazil	Discrimination, misinformation, and poorly designed policies limited access. Discrimination increased service avoidance 6.7-fold. Many lacked providers support and used hormones unsupervised	(6)
33; Costa et al., 2018; Brazil	Quantitative	To investigate HIV-related health needs and access barriers among Brazilian trans and gender-diverse people	Though 63.7% had tested for HIV, many lacked status knowledge, and 71% were unaware of PEP. Stigma and prejudice discouraged testing and care engagement	(64)
34; Jalil et al., 2018; Brazil	Quantitative	To assess PrEP knowledge and willingness to use it among trans women in Rio de Janeiro and identify associated factors	Awareness was low, but willingness was high. About 70% of HIV-negative trans women met PrEP eligibility criteria by CDC and Brazilian Ministry of Health standards	(65)
35; Marcela-Domínguez, Ramírez, Arrivillaga-Quintero, 2018; Colombia	Quantitative	To examine access to preventive, curative, and specialized health services among transgender women in Cali, Colombia	Participants reported limited access, poor living conditions, high out-of-pocket costs, and absence of services for body modification or psychosocial support	(20)
36; Ferreira, Pedrosa, Nascimento, 2018; Brazil	Qualitative	To understand access and comprehensive care dimensions from a gender diversity perspective	Barriers included a heteronormative biomedical model, disregard for chosen names, and lack of specialized services	(66)
37; Zalazar et al., 2018; Argentina	Qualitative	To explore contextual, social, and individual barriers and facilitators to healthcare—especially HIV services—and intervention acceptability	Contextual barriers included long waits; social barriers included stigma from providers and peers. Self-exclusion and anticipated stigma led to self-medication. Peer support facilitated access	(67)
38; Donoso, Nuñez, Parra-Villarroel, 2018; Chile	Qualitative	To explore trans users' experiences with gender identity recognition in the Chilean health system	Systemic disregard for trans identities hindered access. However, care at the national transgender health hospital improved participants' quality of life	(68)
39; Neer, 2018; Argentina	Qualitative	To analyze how health professionals in Greater Buenos Aires received the Gender Identity Law	The law shifted professional discourse from diagnosis to monitoring, from rigid protocols to customization, and from minimizing risks to cost-benefit evaluation	(69)
40; Ritterbusch, Correa Salazar, Correa, 2018; Colombia	Qualitative	To examine health rights violations experienced by trans women in Colombia	Trans women reported systemic violence, from bodily to institutional levels. Discrimination and procedural abuse in healthcare led to service avoidance and risky informal alternatives	(70)
41; Rocon et al., 2018; Brazil	Qualitative	To identify challenges in promoting trans health, diagnosing issues, and proposing SUS-based actions	Urgent needs included continuous professional training, universal access to hormones/silicone, and multidisciplinary care to ensure safe body modification processes	(71)
42; Sousa, Iriart, 2018; Brazil	Qualitative	To discuss trans men's health demands	Needs centered on depathologization, body modification, and outpatient care. Lack of public services fueled commodification. Structural transphobia shaped health inequalities	(72)
43; Braz, 2019; Argentina	Qualitative	To analyze waiting as a key element in current trans experiences	Despite the Gender Identity Law, trans men still faced violence and discrimination. Expanding access and training health workers remain critical demands	(73)
44; Braz, 2019; Argentina, Brazil	Qualitative	To explore ambivalences in trans men's therapeutic trajectories in Brazil and Argentina	Brazilian users faced delays and lacked free services, turning to peers and self-medication. Argentine services were free but both countries showed provider discrimination and stigmatizing discourse	(74)
45; Carrara et al., 2019; Brazil	Quantitative	To map trans/travesti populations' sociodemographic profiles and access to health and body modification services	Neither public nor private systems fully addressed their needs. Most accessed private services; institutional hormone access was rare. Hormones were obtained via prescriptions, peers, gyms, or veterinary clinics	(75)
46; Krüger et al., 2019; Brazil	Quantitative	To estimate hormone use prevalence among Travestis and trans women in the Federal District and identify associated factors	64.5% reported continuous hormone use, mostly estrogen/progesterone, injectable or oral. Most (84%) used hormones without prescriptions. Peer guidance was common; use correlated with race, income, and education	(76)
47; Lovison et al., 2019; Brazil	Qualitative	To understand how Travestis and transsexuals in Chapecó, Santa Catarina, perceive healthcare access and care	Discrimination persists, especially through non-recognition of social names. Access and care fall short of SUS recommendations	(77)
48; Mendes, Jorge, Pilecco, 2019; Brazil	Qualitative	To identify how public health and social assistance policies support Travestis and homeless trans women in Belo Horizonte	Participants accessed SUS and SUAS, but services often reproduced violence. Reported issues included discrimination, lack of privacy, and sexual harassment. Positive interactions were linked to individual providers' attitudes	(78)
49; Monteiro, Brigeiro, 2019; Brazil	Qualitative	To analyze trans women/Travestis' experiences with healthcare, sexual/gender discrimination, and transition/HIV services	Despite some social progress, barriers remain. Use of social name faces resistance. Agency and peer networks help navigate care. Bodily changes involve tension between formal and informal services. HIV services are not prioritized; stigma remains a barrier	(79)
50; Nogueira, Aragão, 2019; Brazil	Qualitative	To analyze current challenges in LGBT access to healthcare	Professionals and users lacked awareness of national LGBT health policy. Users reported prejudice; professionals displayed detachment from LGBT realities	(80)
51; Hanauer, 2019; Brazil	Qualitative	To describe trans people's trajectories in meeting health needs	Key moments included self-recognition and pursuit of body modification. Care networks were individually built and shaped by shared experiences	(81)
52; Soares et al., 2019; Brazil	Quantitative	To investigate factors linked to PrEP refusal among trans women in a major poor Brazilian city	Two latent classes emerged: high PrEP acceptability (91.3%) and refusal (8.7%). Refusal was more likely among older, white, higher-income trans women, and those with URAI with casual partners	(82)
53; Rocon et al., 2019; Brazil	Qualitative	To discuss challenges in accessing the SUS gender affirmation process	Barriers included regional service concentration, social name disrespect, and stigmatizing diagnosis requirements aligned with binary norms	(83)
54; Sevelius et al., 2019; Brazil	Qualitative	To explore how stigma and transphobia affect trans women's access to care and HIV program preferences	Discrimination discouraged HIV testing and disclosure. Participants favored gender-affirming, peer-based, and stigma-reducing interventions combining biomedical and social strategies	(84)
55; Wilson et al., 2019; Brazil	Qualitative	To assess awareness, interest, and barriers/facilitators for PrEP uptake among trans women	Few had heard of PrEP, but most expressed interest. Barriers included fear of HIV diagnosis, low testing rates, adherence concerns, and past discrimination in SUS. Tech-based education was recommended	(85)
56; Valenzuela-Valenzuela, Cartes-Velásquez, 2020; Chile	Qualitative	To describe the work of the Trans Participation Roundtable in the Talcahuano Health Service	Two themes emerged: need for depathologized, quality care; and dialogue/social participation. Collaborative efforts between professionals and trans leaders were deemed essential	(86)
57; Cortes, Araújo, Pinho, 2020; Brazil	Qualitative	To analyze transgender people's access to healthcare	Barriers included provider prejudice, lack of training, and difficulty forming bonds in PHC	(87)
58; Calderón-Jaramillo et al., 2020; Colombia	Qualitative	To identify sexual and reproductive health needs of trans people and guide service adaptation	Barriers included costs, lack of insurance, provider stigma/abuse. Sexual and reproductive health needs needs included trans specific services, such as sensitive transition care, endocrinology, and surgeries	(88)
59; Maschião et al., 2020; Brazil	Quantitative	To analyze non-prescribed hormone use among trans women in seven São Paulo cities	90.7% used hormones, mostly without prescription. Use was linked to sex work, early initiation (< 18), low education, and being travesti. Social name recognition reduced risk of unprescribed use	(89)
60; Guimarães et al., 2020; Brazil	Qualitative	To evaluate PNSILGBT implementation in PHC and nurse knowledge on diversity and homophobia	Lack of LGBT-related training, poor knowledge of PNSILGBT, and unprepared professionals limited trans access to care	(90)
61; Moraes, Silva, 2020; Brazil	Qualitative	To explore concepts, fears, and suggestions regarding humanized trans care in PHC	Participants cited barriers to access and called for improved professional training, collaboration with social movements, and visibility campaigns	(91)
62; Rocha et al., 2020; Brazil	Quantitative	To examine the HIV care cascade among trans women in São Paulo	Among HIV+ trans women: 80.9% diagnosed, 76.6% prescribed ART, 90.3% on treatment. ART uptake was higher among older adults and those registered in services	(92)
63; Oliveira, Romanini, 2020; Brazil	Qualitative	To understand how trans trajectories are shaped by public health policy in interior Rio Grande do Sul	Policy existence alone does not ensure access. Persistent barriers include provider bias, stigma, and lack of welcoming care. Positive provider-user relationships enhanced health promotion	(93)
64; Rocon et al., 2020; Brazil	Qualitative	To problematize the idea that poor provider training alone causes trans discrimination and exclusion	Health worker training reproduced binary, stigmatizing norms. Authors call for trans-inclusive approaches that value lived experiences and challenge normative practices	(94)
65; Longino et al., 2020; Peru	Qualitative	To explore HIV-risk perceptions, trust in trials, and PrEP information sources among trans women sex workers	Participants distrusted drug trials and preferred receiving PrEP information from fellow trans sex workers	(95)
66; Braz, Almeida, 2020; Brazil	Qualitative	To present anthropological reflections on pathways trans people take to access the Transsexualizing Process	Waiting and aging held different meanings. Many avoided formal care due to negative experiences and relied on peers/social media for health guidance	(96)
67; Beltrán, 2020; Colombia	Qualitative	To address education, employment, and health needs of transgender women in Bogotá	Access inequalities affect economic resources needed for education and healthcare. Barriers include ability to pay, mandatory diagnostic requirements, and unsafe transition processes, undermining rights to health and self-determination	(97)
68; Baccarim, 2020; Brazil	Qualitative	To analyze barriers to public health access among trans people in Curitiba	Public health policies face obstacles related to acceptability, accessibility, quality, and availability. Gender norms hinder implementation and access to transgender healthcare	(98)
69; Costa et al., 2021; Brazil	Quantitative	To determine prevalence and correlates of non-prescription hormone use among Brazilian trans women	Younger, less educated participants with unstable housing and combined hormone use more often used non-prescribed hormones. Almost half were not using hormones	(99)
70; Florêncio et al., 2021; Brazil	Qualitative	To discuss transgender people's therapeutic itineraries from the user's perspective	Comprehensive care for transgender people was evidenced through four categories analyzed: low demand for transgender people in health services; (no) use of the social name in the services; care permeated by prejudiced and discriminatory attitudes; and healthcare system and professionals who are not able to address users' health issues	(100)
71; Leite et al., 2021; Brazil	Quantitative	To examine associations between gender discrimination and medical consultations and HIV testing among transgender women in northeastern Brazil	Gender discrimination reduced access to medical appointments and HIV testing significantly. Most participants experienced discrimination	(101)
72; Shihadeh, Pessoa, Silva, 2021; Brazil	Qualitative	To investigate how health services (fail to) provide care for the LGBTQIA population	Health professionals practice heteronormative care, lack training on transgender issues, and often do not use social names, limiting access. Users seek respectful reception and mental health support	(102)
73; Zucchi et al., 2021; Brazil	Qualitative	To analyze PrEP acceptability among adolescent MSM, Travestis, and transgender women	Low PrEP knowledge and acceptability stem from rigid condom-focused prevention and stigma. Successful uptake requires addressing access barriers and discrimination	(103)
74; Sabino et al., 2021; Brazil	Quantitative	To analyze factors associated with ART adherence and quality of life among transgender women in São Paulo	Adherence was high, increasing with age. Quality of life was generally good but reduced by younger age, lower education, comorbidities, substance use, and depression	(104)
75; Cohen, De Tilio, 2021; Brazil	Qualitative	To understand how transsexuals perceive health care	Barriers include lack of information and prejudice from staff and other users. Social name use was mostly respected	(105)
76; Zalazar et al., 2021; Argentina	Qualitative	To explore transgender women's experiences with HIV service scale-up in Argentina	Ethical concerns include autonomy violations, discrimination, disrespect, and poor ART information among trans women living with HIV	(106)
77; Nogueira, Leitão, Silva, 2021; Brazil	Qualitative	To analyze implementation of National Policy for Comprehensive Health for the LGBT Population (PNSILGBT) and access difficulties for Travestis and transsexuals in Parnaíba	Trans and travesti women face prejudice, delays, and resort to self-medication and unsupervised procedures due to lack of respectful, inclusive care	(107)
78; Prates et al., 2021; Brazil	Quantitative	To evaluate dental service use, oral health self-perception, and impacts among transgender people	Dental service use was similar to cisgender people, but transgender participants reported lower satisfaction and higher perceived need for treatment	(108)
79; Silva Filho, Nascimento, Castro, 2021; Brazil	Qualitative	To analyze mental health assistance for trans population at two Psychosocial Care Centers in Brazil	Teams recognize discrimination and marginalization but hold stereotypes; social name use aids reception, but more team training is needed for ongoing care	(109)
80; Clark et al., 2021; Peru	Quantitative	To pilot a social network intervention trial to promote PrEP adherence among transgender women	Intervention group showed trends of higher PrEP adherence and protective drug levels compared to controls	(110)
81; Silva, Tajra, Luz, Sales, 2021; Brazil	Qualitative	To understand therapeutic itineraries of trans population in Timon, Maranhão, Brazil	Participants face intolerance and violence, rely on informal care and self-administered hormones, with poor formal service linkage and continuity	(111)
82; Zapata Pizarro et al., 2021; Chile	Quantitative	To assess Chilean medical professionals' knowledge on transgender health care and regulations	Doctors have limited training but express interest in respectful transgender care and support medical assistance in public health	(112)
83; Jalil et al., 2022; Brazil	Quantitative	To assess use, retention, and adherence to daily oral PrEP and predictors of non-attendance in the Brazilian PrEParadas study	Despite high retention, adherence was low. Gender-affirming environments facilitate PrEP delivery. Socioeconomic disparities affect adherence	(113)
84; Lazcano, Toneli, 2022; Brazil	Qualitative	To discuss meanings around trans-specific care from dialogues with users, doctors, and staff in southern Brazil	Medical residents and trans activists promote trans-specific care despite limited institutional support; professionals partially recognize trans rights and health needs	(114)
85; Miwa, Neves, Therense, 2022; Brazil	Qualitative	To understand daily negotiations about safe sex and health service access among Travestis and transgender sex workers in Manaus	Participants faced discrimination and perceived unprepared professionals, deterring care. Condom use was inconsistent due to client pressure and distrust of PEP	(115)
86; Mota et al., 2022; Brazil	Qualitative	To analyze relationships between health service access and social suffering among trans people	Social name denial and institutional transphobia cause marginalization and distress, despite policies supporting respectful care	(116)
87; Rocon et al., 2022; Brazil	Qualitative	To problematize professional training aimed at removing barriers to trans population health service access	Training is biomedical and hierarchical, limiting user participation. Collaborative learning is proposed to improve attitudes and services	(117)
88; Silva et al., 2022; Brazil	Quantitative	To analyze non-prescription hormone use among Travestis and transsexual women in Salvador, Brazil	Majority use non-prescribed hormones linked to silicone use and body satisfaction. Comfort with genitalia was associated with less hormone use. HIV-positive participants used hormones more	(118)
89; Moncayo Quevedo et al., 2022; Colombia	Qualitative	To review reasons for condom use and non-use among transgender women in Colombia using the IMB model	Participants had knowledge but initial misinformation and social barriers led to inconsistent condom use. Interventions should address these gaps and barriers	(119)
90; Rossi et al., 2022; Brazil	Qualitative	To analyze knowledge, perceptions, care practices, and therapeutic itineraries for STD diagnosis and treatment, emphasizing syphilis, among Travestis and transgender women in Salvador	Knowledge gaps and contradictory perceptions of STDs, especially syphilis, were identified. Two main care pathways highlighted stigma and discrimination in services	(120)
91; Neer, Newton, 2022; Argentina	Qualitative	To explore meanings nurses and social workers ascribe to their practices toward travesti and transgender people	Professionals report discrimination, lack of gender identity recognition, and barriers like insufficient hours and low physician engagement. Some use inclusive practices to improve support	(121)
92; Radusky et al., 2022; Argentina	Quantitative	To examine sociodemographic and psychosocial factors linked to gender identity among people disengaged from HIV care in Argentina	Trans women were younger, more dependent on public insurance, reported higher substance use, poorer provider relationships, and lower ART adherence than cisgender participants	(122)
93; Konda et al., 2022; Brazil, Mexico, Peru	Quantitative	To assess factors associated with long-term PrEP engagement and adherence among trans women in the ImPrEP study	Nearly half had long-term PrEP engagement, positively linked to early adherence and negatively with condomless sex with unknown partners, migration, and Mexican origin. Older age and higher education increased adherence; Peruvian origin and adverse effects reduced it	(123)
94; Oliveira et al., 2022; Brazil	Qualitative	To analyze the therapeutic itinerary of transgender people in a city in Bahia's interior	Participants faced stigma, discrimination, and violence worsened by low education and poverty. Public services were inadequate, pushing many toward commodified, unsafe private care. Barriers shifted care-seeking responsibility to patients	(124)
95; Gomes et al., 2022; Brazil	Qualitative	To identify reasons limiting transsexuals' access to primary health services	Trans people face multiple access barriers to PHC and non-recognition as SUS healthcare users, leading to exclusion and limited rights	(125)
96; Pereira et al., 2022; Brazil	Qualitative	To analyze MSM and transgender women's access to HIV prevention technologies and practices during the COVID-19 pandemic in Curitiba, Brazil	The COVID-19 pandemic increased vulnerabilities, especially for trans/Travestis sex workers who resumed work despite risks. PrEP was used among some	(126)
97; Hernández et al., 2022; Brazil	Qualitative and Quantitative	To discuss strategies and conditions for transgender people's health care access	Access is precarious; services must better meet high demand without blaming users. Travestis have the weakest professional support	(127)
98; Arce-Leonel, Hoyos-Hernández, Ramos-Lucca, 2022; Colombia	Qualitative	To characterize life stories of Colombian health workers experienced in caring for trans women	Barriers include lack of knowledge about trans identities, needs, and stigma in cisnormative society. Health care is not affirming or humanized	(128)
99; Oliveira et al., 2022; Brazil	Qualitative	To understand meanings of being a trans or travesti woman in care provided by the Unified Health System	Prejudice led many to avoid care. Hormonization was essential for gender affirmation; social name use was an important achievement. Self-medication due to lack of trained professionals was common	(129)
100; Cortes et al., 2022; Brazil	Qualitative	To analyze transgender men's itineraries seeking access to the transsexualizing process	Barriers included long waits, costs, and unprepared or disrespectful professionals, hindering adequate support	(130)
101; Borgert et al., 2023; Brazil	Qualitative	To understand the health-disease-care process of trans people and their health service access	Difficulties arise from lack of knowledge on trans issues, low acceptance of gender expression, and discrimination, impacting mental health	(131)
102; Carosella et al., 2023; Peru	Quantitative	To analyze morbidity and health service use patterns	Trans women had suboptimal health coverage. Older age and lower education increased risks. HIV testing was high but PrEP uptake was low	(132)
103; Paiva et al., 2023; Brazil	Qualitative	To understand transsexuals' perceptions of health care network access in a Minas Gerais municipality	Disrespect for social names and professional prejudice limited formal care. Informal networks provided hormone information but posed risks if solely relied on. Formal care was unstable and insufficient	(133)
104; Dias et al., 2023; Brazil	Qualitative	To investigate transgender men's perceptions of gynecological care access and use	Lack of respect and preparation hindered access. Fear of (re)traumatization reduced demand, highlighting need for humanized primary care skills	(134)
105; Gomes et al., 2023; Brazil	Qualitative	To explore transgender people's experiences and demands in primary health care services	Trans people face prejudice, social name disrespect, care refusal, and delays. Care remains cisheteronormative, complicating access	(135)
106; Jesus et al., 2023; Brazil	Qualitative	To understand transgender women's care experiences in public health services	Participants lacked primary healthcare links, experienced institutional violence and prejudice. Suggested improvements include humanized care, hospital accreditation, worker well-being, protocols, and complaint channels	(136)
107; Luca et al., 2023; Argentina	Qualitative	To explore acceptability, perceptions, and recommendations for HIV self-testing implementation among transgender women	Motivations include convenience, privacy, and stigma reduction. Recommendations favor distribution via primary and trans-sensitive centers, affordability, peer support, and clear instructions	(137)
108; Pinto, Saletti-Cuesta, 2023; Argentina	Qualitative	To study COVID-19's impact on transgender people's access to healthcare and hormone treatments in Córdoba, Argentina	Pandemic hindered hormone access, exacerbating inequalities. Trans and travesti-led networks were key to ensuring health rights	(138)
109; Radusky et al., 2023; Argentina	Quantitative	To describe self-reported mental health, substance use, violence experiences, and healthcare access changes among Argentine transgender and non-binary populations after lockdown	Transfeminine participants reported socioeconomic losses and access barriers. Transmasculine and non-binary groups experienced higher psychological distress. Substance use decreased overall. Hormone access was disrupted for half of transfeminine and transmasculine participants. Mental health care access was limited	(139)
110; Barrington et al., 2023; Dominican Republic	Quantitative	To assess associations between various stigmas and HIV treatment outcomes	High HIV care engagement and ART use were reported, but viral suppression was moderate. ART interruptions linked to substance use. Higher enacted stigma was paradoxically associated with better ART adherence	(140)
111; Vissicchio et al., 2023; Argentina	Qualitative	To identify main barriers to inclusive healthcare and analyze specific needs of trans people in Buenos Aires province	Organizational and symbolic barriers, resource shortages and gender mistreatment, impede inclusive healthcare services in hospitals	(141)
112; Amarante et al., 2024; Brazil	Quantitative	To assess if anticipated stigma affects communication between transgender women living with HIV and healthcare providers	Social oppression increased fear of mistreatment, which hindered symptom reporting to providers	(142)
113; Galvão et al., 2024; Brazil	Qualitative	To explore challenges in human milk banks providing chest feeding care to transgender men	Gaps in the educational, institutional and management spheres, associated with personal and social issues, reproduce a pre-conceived cis-normative model and disregard the specific demands of providing chest feeding care for the trans population	(143)
114; Zapata, Hoyos, 2024; Colombia	Qualitative	To describe life narratives regarding healthcare for gender transitions among trans women in Colombia	Participants reported stigma, discrimination, and barriers to preventive and specialized care, leading many to self-manage transitions. The health system lacks gender-affirming readiness	(144)
115; Medeiros, Gomes, Spinelli Junior, 2024; Brazil	Qualitative	To examine links between health service access/use and stress and resilience among Travestis and transgender women in northeastern Brazil	Access was similar across participants, but all faced stress during care. Resilience factors led many to seek care outside public services due to access difficulties	(145)

All reports presented field research, and the majority (71.2%) adopted a purely qualitative approach (Table 4). Across these and mixed-methods studies, the majority did not specify a clear methodological framework, merely describing their design as qualitative (Table 5). Ethnographic, phenomenological, and grounded theory approaches were the most frequently reported among those that specified one.

**Table 5:** Article attributes summary.

**Article attributes**	*n* (%)^*^115 (100%)
**Language**	
English	39 (33.9%)
Portuguese	35 (30.4%)
Spanish	18 (15.6%)
Bilingual	23 (20%)
**Methods**	
Qualitative	82 (71.3%)
Quantitative	27 (23.5%)
Quali-quantitative	6 (5.2%)
**Study population**	
Exclusively Trans, Travestis, and GD people	83 (72.2%)
Transfeminine identity (Trans women, Travestis, or others)	53 (46.1%)
Transmasculine identity (Trans men or others)	6 (5.2%)
Transfeminine + Transmasculineidentity	15 (13%)
Transfeminine + Gender-diverse	1 (0.9%)
Transfeminine + Transmasculine identity + Gender-diverse	7 (6.1%)
Trans (not specific)	1 (0.9%)
Exclusively Healthcare professionals	9 (7.8%)
Mixed Populations (Trans and cis people (living with HIV, MSM, LGB, HC workers)	23 (20%)
Transfeminine identity (Trans women, Travesti, or others)	12 (10.4%)
Transmasculine identity (Trans men or others)	1 (0.9%)
Transfeminine + Transmasculineidentity	6 (5.2%)
Transfeminine + Transmasculine identity + Gender-diverse (non-binary, agender, fluid, or others)	2 (1.7%)
Trans (not specific)	2 (1.7%)
**Health care level**	
Unspecified	65 (56.5%)
PHC + Specialized Care	22 (19.1%)
Specialized care (secondary and/or tertiary)	19 (16.5%)
PHC	9 (7.8%)
**Nature of the institutions conducting research**	
Public	76 (66.1%)
Private	39 (33.9%)
**Financial support**	
Yes	62 (56.5%)
No	32 (27.8%)
Not informed	21 (12.3%)
**First author's detailed field (ISCED-F 2013)**	
Psychology	35 (30.4%)
Nursing and midwifery	24 (20.9%)
Medicine	17 (14.8%)
Sociology and cultural studies	16 (13.9%)
Social work and counseling	6 (5.2%)
Pharmacy	4 (3.5%)
Political science and civics	3 (2.6%)
Dental studies	3 (2.6%)
Others	7 (6.1%)
**First author's narrow field (ISCED-F 2013)**	
Social and behavioral sciences	54 (46.9%)
Health	50 (43.5%)
Welfare	6 (5.2%)
Biological and related sciences	2 (1.7%)
Business and administration	1 (0.9%)
Environment	1 (0.9%)
Journalism and information	1 (0.9%)
**Health goods and services addressed**	
Trans-specific needs (e.g., gender-affirming care, specialized Trans clinics)	29 (25.2%)
General needs (cis and Trans populations)	70 (60.9%)
Both general and trans-specific needs	16 (13.9%)

MSM, Men who have Sex with Men; LGB, Lesbians, gays, and bisexuals. ^*^Percentages refer to the total sample (*N* = 115).

Quantitative (23.48%) and mixed-method studies (5.2%) were predominantly cross-sectional and descriptive, focusing on sociodemographic factors, healthcare access, hormone use, body modification, and HIV prevention behaviors (Table 4). Analytical studies explored associations with HIV care, ART adherence, psychosocial factors, and quality of life. Only one cohort study examined PrEP engagement and adherence, and two intervention studies assessed adherence-promoting and clinical follow-up strategies among transgender women.

Table 5 presents the key characteristics of the included studies. Most were published in English or Portuguese, and over 70% focused exclusively on Trans, Travestis, and Gender-Diverse (TTGD) populations (1–4, 7–8, 11–19, 21–24, 26–27, 29–35, 37–49, 51–55, 57–63, 67–71, 74–76, 79–83, 85–91, 93–95, 97–107, 109–110, 112, 114–115). Smaller proportions examined only healthcare professionals (7.8%) (22, 31, 39, 60, 68, 79, 82, 91, 98) or included more than one sample population (20.0%) (5, 6, 9, 10, 20, 25, 28, 36, 50, 56, 64–66, 72, 73, 77, 78, 84, 92, 96, 108, 111, 113), such as Trans people alongside cis individuals living with HIV, Men who have Sex with Men (MSM), Lesbian, Gay, and Bisexual (LGB) individuals, and healthcare workers.

Nearly half of the studies focused exclusively on transfeminine identities (1–4, 8, 11–14, 16–17, 19, 26–27, 29–30, 34–35, 37, 40, 46–49, 52–55, 59, 61–63, 67, 69, 71, 74, 76, 80, 83, 85, 87–90, 93, 99, 102, 106–107, 110, 112, 114–115).

The healthcare worker population included community health workers; mental health professionals (psychologists, psychiatrists, and therapists); nursing staff (nurses, nursing assistants, and technicians); physicians and medical specialists (surgeons, endocrinologists, gynecologists, urologists, and sexologists); allied health and support staff (social workers, hospital receptionists, midwives, nutritionists, oral health technicians, emergency medical technicians, and physical educators); and professionals in healthcare-related roles (lawyers, journalists, health service coordinators) (9, 10, 22, 28, 31, 39, 50, 56, 60, 64, 68, 77, 79, 82, 84, 91, 98, 113). Three studies did not specify professional categories (66, 108, 111). Most studies (56.5%) did not report the level of care (1, 3–5, 7–15, 19–22, 25, 27, 30, 33–34, 37, 40, 46–47, 51, 54–55, 57–59, 62–63, 67, 69, 71–73, 76–78, 80, 83, 85, 87–93, 98–99, 101–104, 107–109, 112–115).

Contributions from Sociology and Cultural Studies, as well as Social Work and Counseling, played a meaningful role in shaping the literature, as Social and Behavioral Sciences narrow fields (146) accounted for nearly half of the first authors' disciplinary backgrounds (46.9%) (1–2, 4–5, 7–8, 10, 21–26, 28, 32–33, 35–37, 39, 41–45, 48–50, 53–55, 58, 61, 63–64, 68, 72–73, 75–77, 79, 84–85, 87, 89, 91–92, 96–98, 108–109, 111). Health disciplines followed closely, representing 43.5% (3, 11–19, 29–31, 34, 46–47, 51–52, 57, 59–60, 62, 67, 69–71, 74, 78, 80–83, 86, 88, 90, 94–95, 99–101, 103–107, 112–115).

### 3.2 Access to health services and goods

**Figure 4:**
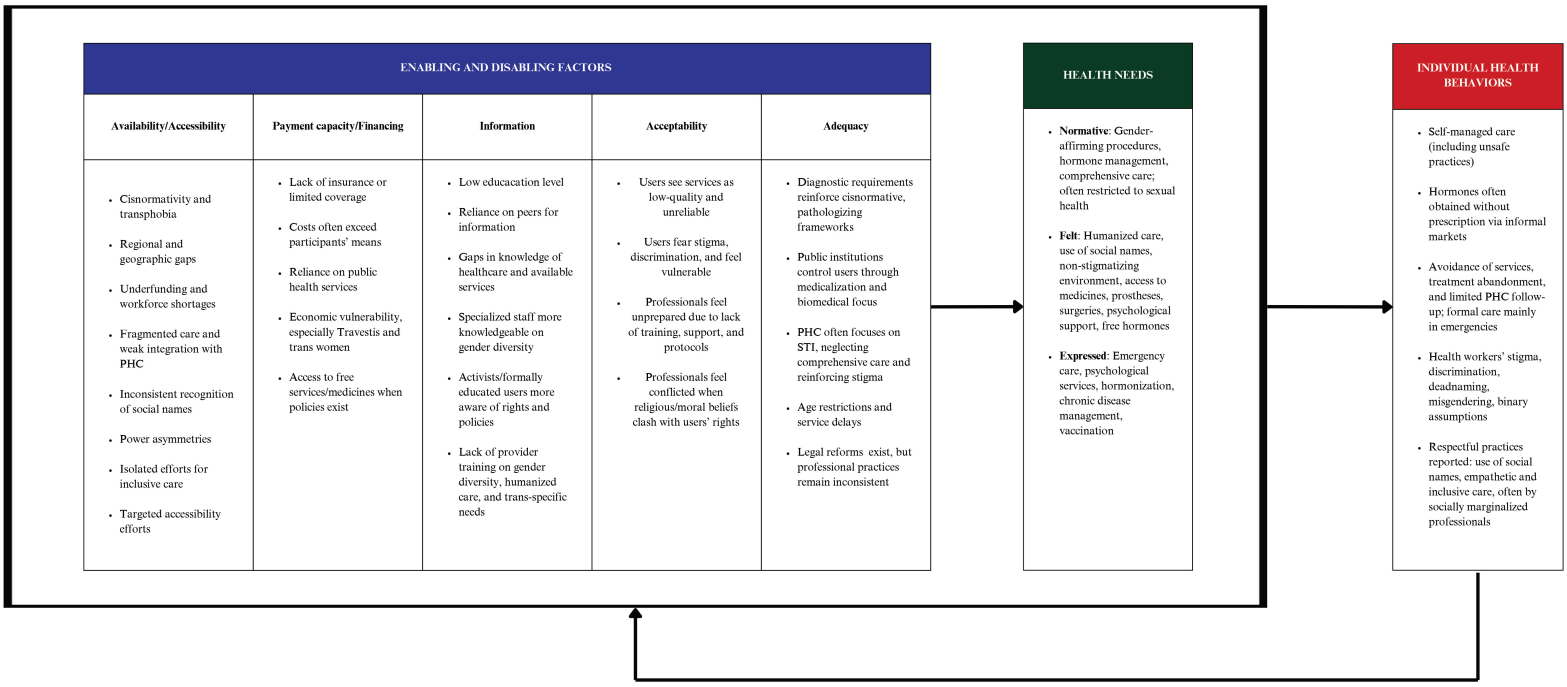
Representation of key findings within the adapted Andersen Behavioral Model. Adapted Andersen's Behavioral Model: incorporating the dimensions of information, acceptability, and adequacy (28, 29) and considering broad health needs (30). STI, Sexually Transmitted Infection; PHC, Primary Health Care.

Individual and contextual factors that enable or hinder access to healthcare for TTGD populations in Latin America are synthesized in Figure 4, which presents the Adapted Andersen Behavioral Model, highlighting the principal findings of this scoping review.

#### 3.2.1 Enabling and disabling factors

##### 3.2.1.1 Availability/accessibility

Availability, similar to accessibility, refers to the presence of health services and goods in the right place and time, considering geographic proximity, transport options, and provider willingness (22, 29). Studies indicated that the availability and accessibility of healthcare for TTGD populations in Latin American public health systems remain fragmented and inadequate. Services across all levels are shaped by systemic cisnormativity, institutional transphobia, regional disparities, underfunding, and prejudice. These challenges are compounded by structural and organizational barriers, which hinder comprehensive, affirming, and timely care. Altogether, these elements operate within a broader social apparatus that reproduces institutional violence, adapting its forms and sustaining its effects across contexts (14, 16, 19, 26, 40, 48, 61, 63, 68, 79, 84, 86, 106, 115).

The availability of healthcare services and goods across the region is characterized by a mix of isolated positive initiatives and persistent systemic obstacles. Many reported non-uniform care due to discrimination or geographic inaccessibility (9, 12, 71, 109). Challenges include scheduling, bureaucracy, unwelcoming or unsupportive reception, physician shortages—particularly in specialized gender-affirming services but also in general care units—and information systems that fail to accommodate social names (49, 58, 66, 68, 104, 105, 114). A study showed a lack of coordination between health systems, universities, and social movements, as well as an absence of outreach promoting service use (61). Institutional denial of gender identity was frequently reported, particularly through the use of legal (rather than social) names and sex assigned at birth (1, 3, 63, 68, 86). “Social names” is a Latin American term referring to a person's chosen name (Brazil, 2016; Argentina, 2012), similar to the notion of “chosen or affirmed names” in global northern contexts.

Public Primary Healthcare (PHC) often lacks structural support and qualified professionals, compromising both the delivery of humanized care—understood here as a welcoming and person-centered approach—and PHC's function as a gateway for TTGD users (3, 4, 11, 18, 26, 31, 38, 48, 51, 55, 95, 103, 115). Users report weak connections to PHC, demanding better integration among providers and users, as well as more welcoming environments (106). Care quality varies by region, with central units having more staff and greater availability of gender-affirming services compared to outlying areas. The limited access to gender-affirming procedures and higher prevalence of stigma in peripheral or rural locations often compel TTGD people to seek private care or clandestine alternatives (24, 48, 103). Despite increased demand for care in Psychosocial Public Care Centers, professional and service capacity to support TTGD individuals remains insufficient across settings (79). Proximity to PHC facilities and ease of transportation further influence service utilization, as TTGD users may avoid distant or non-affirming centers due to anticipated discrimination or inadequate care (115).

Some services are characterized by power imbalances in the relationships between physicians and TTGD users, weakening therapeutic bonds, and discouraging care-seeking. In addition to inadequate professional qualifications, these issues are compounded by institutional neglect (e.g., disregard for users' gender identity, providers remaining silent when users raise sexual or gender diversity–specific issues, and breaches of confidentiality) and a lack of disaggregated data on users' characteristics, as well as the capacity of health services for planning and resource allocation. Care also tends to be predominantly curative rather than preventive (9, 111).

Healthcare was often perceived as prejudiced, violent, and inadequate, marked by misgendering, denial of care, medical coercion, and other forms of institutional violence detailed below (2, 3, 14, 16, 26, 40, 67, 99, 104, 106). Reported barriers included inappropriate hospital ward allocation (1, 3) and prejudice from other service users (99). In Brazil, although TTGD individuals frequently experience injuries resulting from public violence, they rarely seek care in the public health system, perceiving it as inadequate (14). While discrimination in public care is recognized, availability encourages use, especially for gender-affirming procedures and emergencies (49). Reports of severe violations of healthcare rights in Colombia included torture manifested in continuous forced testosterone injections carried out against users' will, as well as degrading psychological and medical practices that pathologize and humiliate TTGD users (40), with older Trans women experiencing abuse more frequently (67).

Delays in accessing services were a significant barrier. In Colombia, emergency care for some Trans women was immediate, but non-emergency services were often delayed, reflecting uneven system responsiveness (35). Similar delays affected other settings, including Brazil's SUS gender-affirming services (6, 7, 15, 28, 51, 99).

Gender-affirming services were characterized by long waiting times in service queues, limited hours that overlooked sex workers' schedules, bureaucratic hurdles such as excessive documentation requirements, overcrowding within Trans-specific services (high demand facing limited availability), poor signage and infrastructure, lack of coordination among specialists, unqualified professionals, unstable supplies of inputs, and high staff turnover (1, 2, 4, 10, 24, 26, 28, 36, 35, 37, 41, 43, 44, 51, 53, 56, 66, 75, 81, 84, 86, 91, 98, 103, 106, 109, 111, 114).

TTGD participants accessed public gender-affirming services primarily for diagnosis, post-operative care, and some body modification procedures, while others opted for private surgeries due to long wait times (45, 97). Conversely, some individuals reported private services as less respectful of gender identity, including chosen names, despite available information (22).

Gaps in national policies persist. The geographic concentration of health services in capital cities further exacerbates inequities for individuals living in rural or remote areas. The main public gender-affirming care services identified were Brazil's Transsexualization Process (PTr) and Argentina's Gender Identity Law (8, 13, 17, 21, 22, 23, 28, 29, 39, 39, 43, 44, 45, 51, 53, 59, 63, 76, 87, 97, 111), both mostly concentrated in capitals, with long waitlists and travel burdens (24, 26, 36, 41, 42, 44, 51, 53, 56, 100, 101, 114). While the Argentine law promoted depathologization and multidisciplinary care, implementation varies (see Section 3.2.1.5) (22, 28, 39, 98, 108, 111). However, despite the law, limited hospital capacity and human resources in the public sector hinder its full implementation (22).

Brazil faces challenges in implementing its National Policy for Comprehensive Health of the LGBT Population (77, 104), and studies highlight significant criticism regarding the standards of PTr implementation (53). PTr hospital services exhibit poor integration with other Brazilian Unified Health System (SUS) services and a regulatory approach to mental health. The focus on procedures and approaches centered on technological devices leads to a division of responsibilities and the trivialization of users' suffering. Moreover, its institutional requirements are grounded in biomedical and cisnormative logics (see Section 3.2.1.5) (21, 23, 29, 42, 63, 64, 97, 100).

The exclusion of hormonization medicines from Brazil's SUS Essential Medicines List restricts access to hospital-based PTr services, limiting availability. Many obtain hormones through private pharmacies, often without prescriptions or using falsified ones, and via informal or unsafe markets, gyms, or other sources (11, 15, 30, 32, 35, 40, 44, 45, 46, 59, 69, 81, 88, 97). Public access is rare, reported as 1.8% (45) and 2.2% (97). Pharmacies frequently serve as both sources and healthcare facilities for injectable hormone administration (11, 32, 35, 46, 88). Their widespread availability, combined with weak PHC support and limited public provision, fosters self-medication and the use of counterfeit or controlled substances such as testosterone, which requires a prescription in Brazil (1, 3, 7, 8, 14, 15, 17, 18, 21, 24, 25, 37, 40, 41, 42, 44, 48, 49, 58, 66, 81, 95, 99, 100, 103, 114). Hormonization regulation remains weak, with no clear protocols for access, distribution, or clinical guidance (8, 94). Hormone availability was associated with better PrEP adherence (83), and recognition of affirmed names functioned as a protective factor against self-medication (59).

Barriers to sexual health services, ranging from pharmacies to specialized healthcare facilities, included discrimination, HIV stigma, cost, inadequate support, and incompatible service hours, particularly for sex workers, while peer support facilitated access and consistent use (5, 6, 76, 90). Despite limitations, STI services, especially for HIV/AIDS, are often the most Trans-competent and structured facilities available, though their centrality reinforces stigma (5, 24, 63, 74, 76). HIV diagnosis availability occurs through public services, NGOs, and private clinics (20). SUS remains the main source of care for many in Brazil, particularly for HIV prevention and treatment (99). ART and HIV tests were generally available during routine visits or hospitalizations (25, 33, 62, 71), but their use is limited by discrimination and provider unpreparedness (62, 71, 83). Self-testing among Trans women was facilitated by privacy and availability, with recommendations for PHC-based distribution and peer support (107). For STIs like syphilis, care involved SUS or private diagnostics in Brazil, but treatment was accessed only through the public system (90). Condoms were mainly accessed through community organizations and healthcare facilities (89).

During the COVID-19 pandemic, the availability of healthcare services in Argentina was significantly disrupted, particularly for individuals not previously linked to specialized services, resulting in reduced access to hormonization as well as general and mental healthcare (108, 109). Informal support networks played a key role in maintaining access despite service interruptions (108). In contrast, in southern Brazil, PrEP, and PEP services remained available, although users experienced increased exposure and heightened risks of serostatus disclosure (96).

The absence of formal institutional support, such as managerial endorsement, clear guidance, and team training for TTGD healthcare, was a consistent issue. Some providers were unfamiliar with appropriate practices, such as guidance on hormone use, and unaware of the local availability of Trans-specific facilities, such as the SUS Transsexualization Process (40, 63, 68, 70, 75, 77, 99). In northern Chile, 77% of healthcare professionals were unaware of Trans-specific care standards, though 96% expressed interest in learning (82).

Multiple studies highlighted that TTGD individuals are often not recognized as legitimate recipients of care (4, 28, 50, 60, 64, 68, 111, 113). Systemic invisibility, the absence of guidelines and research, the prevalence of cis-heteronormativity (services structured around the assumption of cisgender and heterosexual users, excluding the realities and needs of TTGD people), and professional dominance, expressed in hierarchical, physician-centered models that disregard users' autonomy, contributed to the marginalization of transmasculine individuals in lactation care (113). As a result of systemic barriers, many Trans men avoided accessing gynecological care in public PHC and sought private specialized services, relying on the Brazilian SUS only when no alternatives were available, such as during COVID-19 vaccination (104). Invisibility was linked to the absence of specific policies, data collection systems, and protocols that address the diversity of bodies, identities, and experiences of TTGD. For instance, several studies pointed out that transmasculine individuals and non-binary people remain largely invisible in public policies and health services, especially regarding reproductive and sexual healthcare (26, 33, 42, 58, 64, 66, 72, 75, 84, 85, 94, 97, 98, 101, 104, 107, 108, 110, 113).

Inclusive healthcare facilitated access across all care levels (18, 36). Nurses and social workers reported isolated efforts to improve accessibility for TTGD, including providing gender-responsive services, institutional support, safe spaces (characterized by respect for gender identity, confidentiality, and supportive environments), inter-facility collaboration, and staff training (91). However, Trans-specific PHC initiatives were limited to medical consultations, with little multiprofessional integration or connection to higher-level care, and lacked gender-affirming procedures like surgeries (18).

Respect for social names and inclusive provider attitudes were seen as service strengths, alongside calls for professional training and the hiring of TTGD staff to improve care (48). Some promising initiatives emerged, including community-led protocol revisions, collaborative learning between professionals and TTGD users, LGBTQ+ communication workshops, and mandatory rotations at Trans-specialized facilities for medical residents (5, 48, 56, 64, 84), though these remain isolated and inconsistently applied.

##### 3.2.1.2 Payment capacity/Financing

Payment capacity or financing refers to the relationship between healthcare costs and users' ability to afford them (28, 29). The studies indicate that both users' financial means and funding sources (public, social security, or otherwise) are key determinants of access. Limited financial resources and high costs of Trans-specific care are frequently cited as barriers. Consequently, many rely on public health systems due to the unaffordability of private care, despite concerns over organizational issues, quality, and scope of services (1, 6, 7, 14, 21–24, 38, 44, 49, 53, 56, 66, 84, 100, 102). However, service barriers (see Section 3.2.1.1) led some to seek gender-affirming procedures in private or informal settings (6, 7, 15, 28, 51, 99).

Many studies reported that participants had monthly incomes below the minimum wage (25, 35, 59, 62, 69, 88) and lacked private health insurance (13, 92, 102). An exception was observed in Brazil's Federal District, where most participants earned up to four times the minimum wage (46). During the COVID-19 lockdown, financial aid was particularly difficult for accessing healthcare (109).

Limited income was associated with reduced access to hormonization, gender-affirming surgeries, decreased ART adherence, and limited HIV-related care, while higher income and education correlated with consistent hormone use and access to professional guidance (32, 35, 45, 46, 62, 88, 97). Conversely, lower income and engagement in sex work were linked to non-prescribed hormone use and difficulties communicating with providers due to anticipated mistreatment (88, 112).

Economic vulnerability was particularly pronounced among Travestis and Trans women, with many reporting unemployment or underemployment as barriers to consistent care (20, 40, 48, 49, 67, 97). Unemployment increased the likelihood of fearing mistreatment and reduced communication about health needs (112). Homelessness and reliance on sex work were often linked to financial precarity and the need to fund health-related expenses (48, 49).

Healthcare costs, including psychological support, gender-affirming care, and STI prevention tools like PrEP, often exceeded participants' means, leading to unsafe practices (see Section 3.2.3) (5, 7, 9, 15, 20, 23, 24, 40, 51, 58, 97). Affordability shaped participants' perceptions of HIV self-testing (107). In Peru, Trans women were willing to pay only USD 5 for HIV self-tests, well-below the market price of USD 18, indicating that payment capacity was a limiting factor (25).

In countries with national gender-affirming policies, some participants accessed services and medicines free of charge (43). Some users of public gender-affirming services reported out-of-pocket expenses for items not covered, including post-operative ointments, hormones, or travel to other cities (53, 84).

Most studies reported low insurance coverage and limited benefits for Trans-specific needs in both public and private systems, with few exceptions (1, 38, 44, 56, 58, 81, 97, 114). Even participants with insurance often turned to private care to expedite access or compensate for gaps in the public system. Private services were perceived as higher quality, yet financial constraints often made continuous care, and sometimes even occasional care, unattainable (7, 14, 24, 49, 81, 100, 108).

Private health insurance was associated with lower healthcare avoidance (12), with *Travestis* the lowest coverage rates (97) and the highest uninsured rates among individuals with lower educational levels (102). Some participants used general care from private plans but relied on the public system for gender-affirming procedures (e.g., PTr) (47). Uninsured individuals turned to social insurance, public clinics, or fundraising to access private chest surgeries (44). Even within public national healthcare systems, items like hormones or post-operative supplies required out-of-pocket expenses (53, 108); financial constraints hindered travel for care (53).

The economic barriers were further compounded by systemic inequities, including employment discrimination, educational exclusion, and social marginalization, which restrict TTGD people's earning capacity and autonomy, particularly among older Trans women and those in informal housing or unstable living conditions (1, 20, 37, 40, 67, 89).

##### 3.2.1.3 Information

Information refers to the degree of knowledge asymmetry between users and health professionals, impacting communication and healthcare decision-making (29). The studies reveal widespread limitations in access to accurate and comprehensive health information among TTGD participants across diverse settings.

Educational levels varied but were often below secondary education, likely impacting health literacy (3, 4, 6, 13, 16, 24, 25, 30, 35, 42, 46, 47, 49, 55, 59, 69, 70, 92, 81, 102). Low education levels correlate with limited knowledge of healthcare options, use of liquid silicone, low health literacy, and poorer outcomes (27, 30, 33, 34, 83, 93). Trans women with elementary education or less reported higher illness prevalence (102), while higher education was linked to improved communication with providers (112).

Many relied on peers, social networks, NGOs, and community groups for information on hormonization, gender-affirming procedures, and sexual health (3, 8, 10, 11, 14, 15, 19, 24, 32, 37, 44, 46, 49, 51, 56, 65, 66, 75, 77, 89, 94, 96, 97, 99, 103). Transgender and gender-diverse individuals linked to hormone access clinics showed better knowledge of inclusive care and rights (111). The internet and social media were key for disseminating gender-affirming information and fostering community-informed biomedical knowledge (10, 43, 44, 51, 66, 94, 97, 99). Trans men more frequently used online forums for health information than Travestis (97). A lack of knowledge about how to enroll in health coverage was observed among uninsured individuals (35). Uncertainty about healthcare safety healthcare effectiveness was persistent and age-dependent; younger or newly informed individuals exhibited more uncertainty (3, 73, 77), while older participants recognized self-medication risks and emphasized long-term self-care (37). Misinformation and insufficient guidance contributed to unsafe practices, leading to heightened health risks (11, 15, 77).

Information gaps regarding general healthcare, especially STI prevention and treatment, were recurrent. Some users, especially sex workers, demonstrated awareness of STI risks and prevention (65, 85, 89, 90). Many healthcare users had limited or inaccurate knowledge of HIV and syphilis treatment (3, 5, 54, 55, 65, 73, 76, 89, 107). Awareness of HIV prevention was low (71% unaware of PEP) (33), and lower education levels were associated with reduced PrEP knowledge and adherence (34, 83, 93). Notably, a social network-based intervention did not improve PrEP knowledge over time (80).

Knowledge about healthcare rights, relevant policies, and locally available services was inconsistent. While some healthcare users, especially activists or those with formal education, understood their rights (6, 24, 47, 48, 49), others were unaware of their entitlements, national LGBTQ+ health policies, or Trans-specific services (9, 50, 63, 77). Confusion about how to access services further hindered navigation of the healthcare system (63, 75).

Staff in specialized services, particularly HIV/AIDS care, demonstrated greater knowledge of gender and sexual diversity than those in general health services (4, 5, 22, 56). However, some professionals perpetuated harmful practices, such as linking Trans people to STIs and minimizing condition severity, exemplified by remarks from sexual health providers such as “*you only had syphilis*” (68, 90). Most provider knowledge stemmed from personal initiative or post-graduate training, as formal education rarely mandates coursework on gender and sexual diversity (28, 50, 60, 111).

Biomedical and hetero-cisnormative biases in professional training were frequently cited as major barriers to adequate health-related information (22, 28, 64, 87). Users' access to accurate and relevant health information was limited by inadequate provider qualifications in gender diversity, humanized care, and Trans-specific health needs, representing a critical barrier. Providers, mainly in PHC, were unprepared and lacking knowledge about gender identity, hormonization, gender-affirming procedures, and institutional regulations such as the use of social names (3, 4, 10, 14, 15, 16, 18, 19, 24, 31, 38, 40, 42, 47, 50, 51, 55, 56, 57, 58, 60, 63, 64, 68, 70, 75, 77, 79, 81, 85, 87, 90, 98, 99, 100, 101, 104, 111, 113). They misunderstood or conflated concepts like gender identity, sexual orientation, and sexual behavior (56, 58, 63, 64, 68, 85, 79, 90). The knowledge gap ranged from Trans-specific clinical availability to broader topics, including bodily changes related to hormonization and the reproductive health needs of transmasculine people (18, 104, 113). Disparities in informed consent regarding surgical risks were identified, with 80.6% of transmasculine individuals receiving full information compared to 40.3% of transfeminine participants (45).

##### 3.2.1.4 Acceptability

Acceptability refers to the perceptions held by users and providers regarding personal traits and practices, which shape care-seeking and service provision (28, 29). For TTGD users, acceptability is influenced by perceptions of violent, low-quality, unreliable services, long wait times, and unqualified or untrustworthy providers (9, 20, 24, 26, 42, 58, 76, 94, 100, 105, 114). Fear of stigma, misgendering, and discrimination by both healthcare providers and cisgender users emerged as a major barrier to care (1, 3–5, 7, 8, 10, 14, 16, 17, 20, 23, 24, 26, 27, 28–30, 33, 37, 38, 40–61, 63, 66, 68, 73, 74, 76, 77, 81, 84, 85, 89, 90, 94, 95, 99–101, 104–107, 110, 112–115). Such experiences evoked feelings of exclusion, fear, and vulnerability, leading many to delay or avoid care (3–5, 7, 8, 24, 26, 28, 29, 38, 40, 53, 63, 66, 68, 76, 85, 86, 90, 94, 99, 101, 113, 115). These negative experiences are strongly associated with mental health outcomes, including stress, depression, anxiety, and suicidal ideation (20, 61, 66, 79, 101, 113, 115).

Services were often seen as unresponsive to users' needs, reinforcing expectations of mistreatment (61, 69). Insufficient training and weak coordination with social movements led users to feel insecure, disrespected, and invalidated during care, worsening emotional vulnerability (1, 18, 42, 63, 66, 68, 79). Stigmatizing views, such as associating HIV with Trans identities, providers' emotional distance, discomfort discussing health issues, distrust and doubts about providers or procedures, and avoidance of gender diversity topics, contributed to the erosion of acceptability as a fundamental barrier to equitable care (1, 6, 9, 32, 45, 49, 50, 63, 68, 79, 85, 115).

Even in specialized programs like Brazil's PTr or Argentina's Gender Identity Law, dissatisfaction was common when care failed to meet expectations (21, 43, 63, 100). Insecurity arose even from positive initiatives in PHC, where temporary or voluntary staff participation undermined continuity and trust (84). Pathologizing and medicalized approaches led to feelings of violation and loss of autonomy (23, 29, 54, 63). Users opposed pathologization but feared losing access to services if diagnostic criteria were eliminated (42, 44). TTGD participants reported self-perceptions of oral health marked by shame and anxiety compared to cis people (78). The dissatisfaction and insecurity extended beyond PHC services to specialized contexts.

Satisfaction with body-affirming procedures was reported (2, 17), even when industrial silicone was used (30). Some users considered informal procedures safer when performed by health professionals (49). Transmasculine users expressed insecurity about procedures like neophalloplasty (7) and dissatisfaction with public hospital chest surgeries, citing poor aesthetic outcomes and lower prioritization (43). Hormone use could also bring frustration due to unmet expectations (10), and self-medication fostered a sense of personal accountability for outcomes (15).

Professionals often feel unprepared for gender-affirming care (8, 56, 61, 68, 73, 100, 106), including managing hormonization, navigating bureaucracy, and confronting internal biases rooted in pathologizing views (9, 61, 68, 100). Many professionals lacked recognition and acceptance of Trans identities (50, 101), often influenced by cisnormative biases, such as the belief that Trans men would not wish to breastfeed (60, 113). Religious and moral beliefs, along with a lack of willingness, were identified as barriers when conflicting with users' rights (6, 113). Nurses reported that the way *Travestis* were treated by healthcare staff was central to their care (31). Greater exposure to gender diversity was consistently linked to reduced stigma and transphobia among providers (68).

Some professionals expressed prejudice and discomfort in discussing LGBT health, even though they intended to provide respectful and welcoming care (60). Others reported feeling pressured to prioritize medical reports required for gender-affirming procedures. Additionally, some noted that the visible presence of Trans users in hospitals raised tensions around the use of social names and discrimination (64). Some workers attributed discrimination mainly to other users or within TTGD communities (68). Stereotypes, such as assuming Trans or Travesti users are inherently defensive, undermined empathy and created additional barriers to acceptability (91). Some workers feared backlash from cis healthcare users when providing equitable care to TTGD users (79). Professionals acknowledged that colleagues' resistance to policies and users' identities hinders access (68). While most providers expressed supportive views, a minority admitted discomfort, reflecting underlying stigma (82).

Regarding the acceptability of STI prevention and treatment, PrEP and HIV self-testing were valued for promoting autonomy and were accepted as self-care strategies. Concerns about condom use, side effects, effectiveness, daily dosing, and fears of emotional distress or violence were identified (5, 25, 54, 73, 85). Stigma, distrust, and insufficient or unclear provider communication related to PrEP, PEP, and HIV care hindered uptake and adherence (37, 54, 76). PrEP acceptability varied due to concerns about cost, efficacy, side effects, and adherence (5, 52). In some cases, knowledge gaps and systemic mistrust led to refusal of care (52, 102). Some healthcare users expressed skepticism toward PrEP, associating it with pharmaceuticalization, Trans necropolitics, and distrust in the medical-industrial complex and the state's commitment to Trans health (65, 73), including concerns about the motives behind international trials and safety (65). Another study reported that PrEP was linked to protection and sexual empowerment (25). Positive experiences were associated with respectful care (9, 35, 110) and trust-building interventions, such as community-based PrEP programs (80).

Guilt and responsibility were common when seeking STI care, and treatments such as benzathine penicillin were described as painful yet necessary (90). Trained professionals were viewed as essential to ensure privacy, trust, and adherence support (5, 25). Still, distrust in confidentiality for HIV testing existed (33, 34). Cell phones were reported as facilitators of HIV prevention and care (25). Although less frequent, positive experiences in affirmative care settings involved well-trained providers who were sensitive to gender diversity, which enhanced users' autonomy (10, 37, 43, 63, 105, 106, 111). Users preferred public clinics with specialized Trans or sex worker services, as well as Afro-Indigenous spaces, due to feelings of safety and inclusion (6, 14, 18, 19, 43, 63, 84). Recognition of social names and pronouns promoted feelings of security and respect among users (4, 37, 99). Experiences of attentive care led some users to feel fortunate (4). The inclusion of Trans healthcare workers or peer promoters enhanced acceptability by ensuring confidentiality, respectful support, and understanding, particularly in sexual health services (37, 54, 73, 107).

Users highlighted that improving acceptability requires humanized care, hospital accreditation within national policies (e.g., PTr), investment in staff wellbeing, and clear protocols with accessible reporting systems. They stressed that depathologization advances are vital to reduce transphobia, affirm identities, guarantee rights, and foster trust and engagement with healthcare (106). Community-based care with active social participation was seen as crucial to shaping care models (56). Equity, justice, and the inclusion of Trans professionals were viewed as essential for building trust and improving care (41, 48).

##### 3.2.1.5 Adequacy

Adequacy, referring to the relationship between how resources are organized to receive users and users' ability to adapt to these factors, as well as their perception of appropriateness (28), remains limited for TTGD populations. Many qualitative studies reveal that access to gender-affirming care is conditioned by medicalization and cisnormative frameworks, notably the requirement of diagnoses such as “gender dysphoria” or “transsexualism,” which reinforce binary pathologizing norms and validate only those fitting the “true transsexual” model (7, 24, 44, 53, 62, 64).

PHC frequently focuses on STI prevention or treatment, neglecting comprehensive approaches and reinforcing stigma that predominantly associates Travestis and Trans women with sex work and STIs (24, 30, 31, 68, 94, 95). As a result, these services often exclude social groups (seen Section 3.2.1.4) (95).

Public health institutions in Latin America often operate within cisnormative biomedical frameworks that regulate users' bodies and identities (8, 23, 28, 29, 40, 41, 63, 64, 67, 87, 89, 108). Medicalization and pathologization persist through mandatory psychiatric diagnoses for gender-affirming care, undermining self-determination and bodily autonomy (7, 10, 17, 22, 23, 29, 42, 44, 49, 53, 54, 57, 67, 70, 86, 90, 97, 111). In Argentina, Brazil, and Colombia, services maintain rigid diagnostic requirements for gender-affirming procedures, with some still relying on outdated or pathologizing terms such as “transsexualism,” which remains required by Brazil's PTr despite its removal from ICD-11 (7, 8, 10, 17, 22, 24, 29, 42–44, 53, 63, 87, 108). Psychiatric and psychological assessments, often mandatory and tied to prolonged psychotherapy or hormonization, are commonly prerequisites for gender-affirming procedures, particularly in PTr services, functioning more as gatekeeping than support (7, 8, 10, 17, 24, 28, 29, 44, 53, 63). Requirements such as PTr's biweekly group therapy in centralized facilities further restrict access (63). These institutional demands reproduce biomedical logics that constrain autonomy and selectively validate identities (64, 66, 108), disproportionately affecting Trans men and limiting the recognition of individual needs (108, 114).

Although Argentina's 2012 Gender Identity Law eliminated diagnostic requirements, professional practices remain inconsistent. Some sought assurances to preserve clinical authority, perceiving the law as constraining professional autonomy (39), and still require mental health consultations or social work approval based on the “gender dysphoria” diagnosis (22). Moreover, respect for social names is not always guaranteed, further undermining the adequacy of care (66).

Age restrictions posed barriers to accessing services and goods, with younger Trans women in Brazil seeking non-prescription hormones due to an 18-year age limit, which undermined safe and adequate gender-affirming care (59). A similar age restriction for HIV self-testing limited access for younger users in Peru (25).

#### 3.2.2 Health needs

Data were analyzed using a conceptual framework that classifies health needs as normative, felt, expressed, or comparative (28, 30). Participants reported that their body modification needs were not recognized by public policies, and argued that hormones should be freely provided (8, 9).

Only seven qualitative studies reported normative needs, defined by technical or professional standards. Gender-affirming procedures were framed as such (2), though access often required additional normative criteria (see Section 3.2.1.5) (64, 67, 111). One study noted professionals' recognition of the need for hormone management and comprehensive, welcoming care (84). STI prevention and treatment were also cited as normative, but many professionals restricted care to sexual health, overlooking broader needs (24, 90).

Resources perceived by users as necessary, called felt needs, were discussed in 28 qualitative studies. These included access to respectful, specialized public care, medicines (e.g., minoxidil and hormones), prostheses, surgeries, guidance on usage, specialist consultations, and ongoing multidisciplinary support (2, 7, 17, 18, 21, 41-43, 55, 66, 81, 84, 94, 99, 101). Psychological support was frequently emphasized (7, 18, 42, 58, 72, 81, 90, 94). Humanized care—using social names, active listening, non-stigmatizing environments, and welcoming reception—was highlighted across studies (41, 61, 72, 84, 90, 94). Free access to hormones, especially via Brazil's SUS, was a recurrent request (8, 41). Participants also indicated the need for comprehensive services, including PHC (cholesterol, weight monitoring, and organ function), diagnostic exams and tests, and sexual/reproductive health services (birth control, gynecology, urology, fertility, abortion care, and STI prevention/treatment) (7, 9, 18, 24, 42, 54, 58, 81, 90, 94, 99).

Specific needs linked to aging were reported, especially among those over 40, reflecting previous experiences with informal procedures and a need for emotional, economic, and social stability (67). There was a strong appreciation for non-pathologizing, inclusive services led by qualified professionals offering individualized care beyond binary, cisnormative models (41, 48, 56). One study reported that 71.2% of participants indicated little or no need for medical treatment (71), while another found worse oral health satisfaction and greater perceived dental needs among Trans people (78). Many felt needs arose from institutional neglect, lack of provider qualification, and unequal access. Participants stressed the urgent need for respectful, equitable, and socially just care, especially for vulnerable groups like favela residents and unhoused individuals (9, 44, 48).

Expressed needs are those linked to active help-seeking. They were explicitly described in four studies. One study found that common demands in PHC included hormonization, chronic condition management (e.g., diabetes, hypertension), and vaccination (105). Other needs included access to hormones and surgeries, support for silicone-related complications, non-cisnormative gynecological care, and reproductive services for transmasculine users (111). Only 23% of Trans women sought emergency care, and 21% accessed psychological services in the past 6 months (35). Another study reported that many participants also sought psychological support, but only 34.8% received professional help during gender-affirming procedures (97).

#### 3.2.3 Individual health behaviors

According to Andersen (26), individual health behaviors refer to how users and providers engage with or avoid healthcare services and goods. This study expands that concept to include broader therapeutic strategies beyond formal care, such as self-medication, adherence, service use, alternative care-seeking, and the behaviors of healthcare professionals in providing care (28).

Facing structural barriers, stigma, and discrimination, TTGD people in Latin America often resort to self-managed health strategies. Some reported having to educate providers about their own care (32). Self-medication was common, especially during hormonization, involving home remedies and unsupervised hormone use (1, 3, 7, 8, 10, 11, 14, 17–19, 21, 23, 24, 26, 37, 38, 40–42, 44, 47, 49, 51, 53, 55, 58, 66, 70, 75, 77, 81, 94, 95, 97, 99, 100, 103, 114, 115). Hormone use rates ranged from 52.5% to 96% lifetime and from 45% to 90.8% current (30, 45, 46, 59, 69, 71, 74). Hormonization often began before age 18 and without medical supervision (11, 35, 74, 88); self-medication rates ranged from 64 to 87% (45, 59, 69, 88). Use of high doses without medical guidance was common, leading to adverse effects in some cases (8, 10, 21, 23, 24, 40, 44, 51, 97, 103). In Lima, only 11.8% accessed hormones via prescription, 5.9% received pharmacist recommendations, and 65.9% relied on friends (11). These practices were mainly driven by the limited supply of gender-affirming hormones in public health systems (see Section 3.2.1.1) (94, 97), a situation worsened during the pandemic as users turned to alternative sources and informal support (96).

Body modification practices were widespread. In Argentina, 92.7% of participants reported undergoing procedures (13); in Brazil, 95% had engaged in body modification; breast augmentations were mainly private (42.1%), and breast construction often occurred in clandestine settings (58.1% of Travestis, 23.8% of Trans women) (45). A Colombian study reported that 46.5% of participants had undergone filler injections in non-clinical settings (15).

Industrial silicone injections, often performed by peers known as “bombadeiras,” were frequently reported, especially among Trans women and Travestis (1, 14, 15, 19, 23, 24, 26, 30, 37, 40, 41, 51, 77, 81, 94, 95, 97, 100, 103). Studies reported industrial silicone use ranging from 12.9% to 55.6% (71, 45, 30); however, only 46.2% sought medical care for complications from the procedure in Brazil (30). Structural and economic vulnerabilities (see Section 3.2.1.2) were linked to industrial silicone injection use (30). Homemade binders also emerged as an alternative body modification strategy (96). Industrial silicone use and self-medication with hormones led to serious health complications, such as liver damage, cancer, abscesses, and even death (23, 24, 30, 40, 81).

Avoidance of health services was widespread and included treatment abandonment, delays in seeking care, and lack of PHC follow-up. These behaviors were driven by negative feelings from prior experiences, leading many to forgo care, even for severe conditions (see Section 3.2.1.4) (1, 3, 4, 14, 16, 18–20, 22, 23, 26, 28, 38, 40, 44, 53–55, 57, 70, 72, 77, 79, 81, 84–86, 90, 94, 95, 97, 99, 101, 104, 106, 108, 111, 114, 115). In Argentina and Brazil, 40.7 and 43.2% of Trans women, respectively, avoided care due to concerns related to their gender identity (12, 32). In the Southeast region of Brazil, only 32% of Trans participants and 14.5% of Travestis used public health services (97), while in a capital in the same region, 37.7% of Trans women had not visited healthcare services in the past year (69). Although 68.5% were enrolled in PHC across Northeast Brazil, only 33% relied on it regularly (71).

Formal services care was often sought only in emergencies or as a last resort (1, 3, 9, 14, 18, 19, 37, 40, 47, 55, 106). When searching for care, gender-affirming clinics (mainly for hormonization or surgeries) and STI clinics were preferred (6, 17, 19, 63, 70, 75, 106). In Cali, 25% used pharmacies and 61% accessed clinics or hospitals (35). In Peru, most participants (80.1%) attended clinics regularly, a pattern linked to older age and unemployment (102).

To navigate healthcare and reduce reliance on formal systems, many, particularly sex workers, sought support through peers, social media, or community networks (49, 56, 97, 111, 114, 115). Some attended appointments alone after negative experiences with companions (28). Others used strategic performances, such as “feminine docility” or “middle-class behaviors,” and leveraged activism to navigate public services (49, 56). In settings enforcing binary, pathologizing norms, users often exaggerated gendered behaviors or concealed personal desires to meet institutional expectations and obtain affirming care (53, 64). Alternative care paths were pursued, including Afro-Indigenous religious practices and home remedies, particularly when public care was inaccessible or invalidating (14, 19, 97).

HIV prevention behaviors varied across contexts. Condom use was uncommon with regular partners (89) and influenced by stigma, trust, and financial or client pressures in sex work (3, 22, 27, 89). Most participants had been tested for HIV (25, 33, 34, 102), yet 5.9–6.8% avoided testing due to fears of stigma, breaches of anonymity, or discrimination (33). Participants living with HIV reported challenges with ART adherence (3, 67), although adherence levels were generally high and improving.

Health workers' behaviors strongly influence access to and quality of care for TTGD populations. Pervasive stigma, discrimination, and disrespect were frequently reported, particularly through refusal to use chosen names and pronouns, even when legally mandated, such as in Brazil's SUS (3, 4, 6, 9, 14, 16, 18–20, 22–24, 26, 28, 31, 36–40, 42, 44, 47–49, 53–57, 60, 70, 72, 81, 85, 86, 90, 95, 98, 99, 101, 103–106, 111, 114, 115). Pathologizing attitudes and entrenched biomedical hierarchies also persist (see Section 3.2.1.5) (2, 10, 22, 23, 28, 39, 40, 44, 53, 58, 63, 64, 86, 87, 103, 111).

Pathologizing practices persist among providers, mostly physicians, even in Argentina, where depathologization is policy (see Section 3.2.1.5) (22, 44, 91, 111). Nurses and social workers often delivered more compassionate care, resisting both medical control over users and their professional subordination to biomedicine (91).

Denial of general care, especially in PHC, is common. Refusals include hormone prescriptions, follow-ups, gynecological services, and other preventive care (7, 18, 30, 51, 53, 56, 58, 86, 91, 98, 103–105, 108). Tensions also arise when providers reject high hormone doses self-administered by users (8).

Discriminatory practices by providers span all healthcare levels, including private community pharmacies and specialized services (see Section 3.2.1.4), and often involve verbal abuse, inadequacies in gender-affirming language and communication such as misgendering, deadnaming, and refusal to acknowledge chosen names, as well as binary assumptions, denial of care, stereotyping, HIV stigma, and religiously motivated transphobia, reflecting systemic hostility (1, 3, 4, 9, 12, 13, 14, 16, 18–20, 23, 24, 26–30, 32, 33, 36, 37, 40, 42, 44, 48, 50, 54–59, 60, 63, 65, 68–70, 72, 75, 76, 79, 82, 85, 86, 90, 91, 94, 95, 97–99, 101, 103–106, 108, 111, 112–115). Discrimination was strongly correlated with avoidance of healthcare services (32).

The lack of humanized care, trust, and continuity in user–provider relationships disproportionately affects TTGD people, especially those engaged in sex work (see Section 3.2.1.5) (1, 4, 5, 31, 50, 57, 79, 98). Discrimination intersects with class, age, and place of residence, with younger users often treated with less respect (9). In PHC, some physicians avoid physical exams with Trans users, relying solely on self-reporting, while specialized service providers show less discomfort (3). Such practices have normalized exclusion and poor-quality care, particularly in PHC (31, 47, 53, 55, 57, 79, 90, 95, 98).

Nevertheless, isolated instances of respectful care exist, including empathetic approaches and inclusive participatory practices, often led by professionals from socially marginalized groups (22, 38, 48, 56, 70, 75, 79, 84, 108). However, these actions frequently coexist with ongoing discrimination within the same services (75, 79, 108).

## 4 Discussion

### 4.1 Trends in knowledge production and regional inequities

Knowledge production on TTGD health access in Latin America has followed patterns that reflect structural, linguistic, and political determinants. The concentration of publications after 2007 corresponds not only to expansions in public policies on gender identity but also to the delayed recognition of TTGD health needs within research systems historically oriented toward cis-heteronormative priorities. This temporal pattern suggests that TTGD health became “researchable” only once broader sociopolitical shifts rendered these populations institutionally visible. Additionally, studies show a growing trend, with most publications emerging in the last 6 years. This reflects increasing academic interest, likely linked to the heightened public visibility of LGBTQIAPN+ communities (147) and the implementation of national health policies (12–14).

The uneven representation of TTGD subgroups, with the literature heavily focused on transfeminine experiences while transmasculine and gender-diverse populations receive comparatively little attention, may reflect historical research priorities, greater social visibility of transfeminine people, and methodological challenges in recruiting transmasculine or gender-diverse participants (148).

Research was also found to be unevenly distributed across Latin America, with Brazil's dominance limiting region-specific policy development. The absence of studies from several countries highlights substantial gaps in regional research production and dissemination. While academic dissemination is multilingual, English and Portuguese prevail over Spanish, the region's most spoken language (149). Although English is official only in Guyana and Belize (absent from this review), its dominance marginalizes research in Spanish, Portuguese, and Indigenous languages, reflecting epistemological coloniality, the anglophone bias of Brazil, and the concentration of knowledge in the Global North. Future studies should explore how linguistic and geopolitical hierarchies shape knowledge production and dissemination.

The current evidence base, comprising mostly qualitative studies followed by descriptive quantitative designs, offers important insight into lived experiences but limits the possibility of comparative or system-level analyses. The scarcity of interventional research also indicates that the field has advanced more in documenting inequities than in testing solutions. This pattern is consistent with Lacombe-Duncan (150), who found that although most peer-based interventions reported positive outcomes, few explicitly incorporated gender affirmation, and most focused primarily on HIV, reinforcing the need for peer-led approaches that address holistic health needs and structural barriers to care.

The high predominance of first authors from Nursing and Medicine indicates a concentration of epistemic perspectives and limited engagement from other health fields, such as pharmacy and dentistry, with a near absence of contributions from disciplines like physiotherapy, nutrition, and related areas.

### 4.2 Structural and political context shaping TTGD health

This review considers how recent political shifts, marked by authoritarian, neoliberal, and anti-gender agendas, undermine health rights and research related to TTGD populations. Far-right governments in Brazil (2019–2022, under Bolsonaro) and Argentina (2023–present, under Milei) have advanced an anti-Trans agenda under the guise of austerity and moral conservatism. These dynamics echo global trends in late capitalism, such as the Trump administration in the USA, whose influence has emboldened anti-rights movements across Latin America. Such contexts promote regional policies not only to restrict access to care but also to constrain the development and funding of inclusive public health research, reinforcing systemic invisibility and and exclusion (151, 152).

The literature spans multiple historical periods, with most studies published in the past decade. This temporal distribution is crucial for understanding the persistence of pathologizing narratives and their entrenchment in national systems. While legal advancements in some countries suggest progress toward depathologization, biopolitical practices and systemic barriers still demand urgent policy revisions, updated clinical protocols, and care centered on autonomy, dignity, and Trans rights (151, 152).

In Brazil, for instance, the outdated classification of PTr services persists despite the ICD-11 (153) removing “transexualism” from the chapter on mental and behavioral disorders and replacing it with “gender incongruence.” This regulatory lag has delayed policy reform and impeded the transition toward a less pathologizing framework in the public health system, while private providers are already aligned with the new classification (154). In contrast, Argentina's Gender Identity Law eliminated the requirement for medical or psychological diagnoses for gender-affirming care (13). Nonetheless, implementation remains inconsistent, and clinical practices often fail to meet the law's objectives. Recent political changes under the Milei administration have raised concerns about potential reversals of gender-related rights, underscoring the importance of continued vigilance (152). Although the law constitutes a regional milestone, Gonzales (155) noted the absence of official data, which hampers evaluation of its effectiveness and reflects broader deficiencies in state monitoring.

This review has revealed that gender-affirming healthcare in Latin America often operates within biopolitical frameworks that reinforce cisnormative and binary norms, regulating TTGD bodies via medicalized diagnoses. These medical categories serve as instruments of institutional control beyond care access (156, 157). Despite structural precarity from transphobia, cisnormativity, and underfunded systems, TTGD communities resist through peer networks, self-care, and advocacy, asserting agency and creating alternative care pathways. While such strategies reflect resilience and autonomy, potential risks associated with unsupervised practices underscore the urgency of integrating peer networks and Trans professionals into formal systems to ensure culturally responsive, affirming, and safer care models.

Persistent structural and organizational challenges in Latin American healthcare systems affect both cisgender and TTGD users, including limited service availability, workforce shortages, fragmented care, long waiting times, underfunding, regional disparities, and poor coordination across levels of care, which exacerbate health inequities and service avoidance (15, 16, 158).

### 4.3 Health barriers through the lens of the conceptual framework

Theoretical insights from Lugones (159, 160) and Vergueiro (161) help illuminate how these system-level barriers are historically and structurally produced. Lugones' concept of the “coloniality of gender” and Vergueiro's analysis of cisnormativity in Latin American contexts revealed how colonial, Eurocentric constructs of gender continue to shape healthcare systems through the imposition of binary and cisnormative norms. Lugones' concept of the “coloniality of gender” and Vergueiro's critique of cisgenderism as structured by pre-discursivity, binarism, and permanence expose how dominant epistemologies naturalize gender as biologically fixed and delegitimize non-normative identities. The following sections are structured by access dimensions using this lens.

#### 4.3.1 Availability/accessibility and adequacy

The studies revealed a marked underrepresentation of PHC as a research setting. Gonçalves (162) noted limited research on LGBTQIA+ access to PHC, despite its central role in Brazil's public health system, and identified persistent cis-heteronormative norms as barriers to inclusive, gender-affirming care. Such biomedical dynamics dominating the organization of health systems are overrepresented in the production of knowledge, perpetuate structural exclusion, and sustain unmet health needs. Similar issues have been reported in high-income countries. Across the USA, UK, Australia, Canada, New Zealand, France, and Sweden, Trans and non-binary people frequently face discrimination in PHC, including limited provider knowledge, strained interactions, and unwelcoming environments, highlighting the need to reorient PHC toward equity-based, affirming models (163).

The findings point to significant gaps in both gender-affirming and general health services in Latin America. Care is often fragmented and insufficient, shaped by systemic cisnormativity, transphobia, funding limitations, and inadequate provider qualifications, which reinforce institutional exclusion. The geographic concentration of Trans-specific services in capital cities presents a major access barrier. TTGD health services in North America, Europe, and Oceania are largely urban-centered, with access further constrained in rural and suburban areas. While rural living is not inherently harmful, structural barriers and stigma from healthcare services in these regions sustain persistent health inequities (164).

In line with these structural gaps, Boldrin et al. (165) reviewed the advances and ongoing challenges of the Brazilian PTr. The expansion of gender-affirming healthcare access for Trans people has emerged with persistent weaknesses, including unprepared healthcare professionals, social stigma, pathologizing clinical diagnoses, and limited hospital coverage. They underscore that improving access and addressing stigmatizing practices are essential to ensuring inclusive, biopsychosocial care.

Access to medicines, particularly hormonization and technologies like PrEP and PEP, was widely discussed in the literature. The lack of standardization and the unavailability of public provision for hormonal medicines often forces individuals to rely on informal or clandestine sources, primarily due to financial barriers. Advancing the inclusion of hormones in national lists of essential medicines across Latin America is therefore imperative. In Argentina, where hormonal medicines are standardized, decentralizing distribution remains a challenge. Brazil needs to integrate hormone use into the broader pharmaceutical care system, extending beyond the current hospital-based model.

Pharmacies emerged to occupy a significant and complex position in the healthcare continuum for Trans people, often serving as accessible points for hormone access amid broader barriers to other health services. However, the role and qualifications of pharmacists, potential key actors in medicine access, have remained underexplored, while the training and practices of psychologists, nurses, social workers, and physicians have been discussed under “professional knowledge,” “acceptability,” and “individual health behaviors.” Redfern and Jann (166) emphasized the central role of pharmacists in Trans healthcare, highlighting their capacity to provide gender-affirming, unbiased care, including pharmacotherapy management and laboratory monitoring. Pharmacists' roles in counseling, hormone support, and preventive care underscore their potential to offer culturally competent services (167). However, these observations are primarily drawn from North American studies, and evidence from other regions remains limited.

#### 4.3.2 Affordability/financing, information, and acceptability

Financial factors, including income, employment, and insurance, emerged as key determinants shaping healthcare access for TTGD people in the region. These findings resonate with broader patterns of socioeconomic inequality identified as major barriers to healthcare access across Latin America, where vulnerable groups face compounded disadvantages (168). Such evidence underscores the inequities in access and highlights the pressing need for public, qualified, and accessible healthcare services specifically tailored to the TTGD population.

This study revealed a persistent lack of information for both TTGD populations and healthcare professionals, contributing to informal practices and reinforcing barriers to care. Research on healthcare information from providers' perspectives remains limited, leaving critical aspects of access poorly understood. Acceptability is further constrained by stigma, provider unpreparedness, and systemic obstacles, highlighting how structural and interpersonal factors intersect to limit equitable care.

The lack of professional qualifications and institutional biases in biomedical care reinforce these hierarchies and marginalize TTGD people by privileging cisnormative logics. As such, reliance on informal networks is not only a survival strategy but also an epistemic repositioning that resists colonial frameworks and reimagines healthcare beyond binary and pathologizing paradigms.

#### 4.3.3 Health needs

The scarcity of comprehensive and multidimensional data on TTGD health needs reflects a significant epistemic gap, limiting the development of informed, equitable, and context-sensitive healthcare strategies. This deficit is particularly concerning because it translates into persistent misalignment between the services provided and the actual needs of TTGD populations. Users' felt and expressed needs often remain unrecognized or inadequately addressed, highlighting how institutional biases, professional unpreparedness, and cisnormative frameworks perpetuate inequities. Addressing these shortcomings is therefore essential for designing interventions that are both technically sound and socially responsive.

#### 4.3.4 Individual health behaviors

Users demonstrate significant autonomy, resilience, and agency in navigating care and body modification outside formal systems. However, this often occurs in contexts of risk due to the lack of gender-affirming, competent, and respectful services. Stigma, discrimination, and pathologization by healthcare professionals remain significant barriers to equitable TTGD healthcare. The findings revealed predominantly negative professional behaviors, with few positive exceptions, reflecting a failure to provide culturally competent, gender-affirming care. Discrimination in healthcare settings contributes to adverse health outcomes and reinforces cycles of control and surveillance within cisnormative systems. One of the main results of these barriers was the avoidance of formal health services.

Prior reviews identified barriers to Trans people's access to the Brazilian public system, including discrimination, inadequate care, and inadequate professional qualifications (2, 23, 94). Lima et al. (5) confirmed these Brazilian issues, noting transphobic violence, rights violations, and reliance on alternative care strategies. Transphobia erodes healthcare trust, leading to care avoidance, particularly in rural areas (164).

### 4.4 Regional and global parallels

Progress in the recognition of LGBTQIAPN+ rights across Latin America is uneven. Countries such as Argentina and Brazil have achieved notable legal and social advances, including the recognition of social name rights, same-gender marriage and adoption, the prohibition of conversion therapies, and access to gender-affirming services. According to Corrales, these gains have been facilitated by active social movements, supportive political institutions, and relatively higher income levels. However, economic resources alone are insufficient to explain progress, particularly in contexts with strong conservative religious influence. In contrast, limited political support, weaker social movements, lower economic resources, and conservative religious pressures have hindered progress in other countries (169). This uneven development likely affects both the recognition of rights and local scientific production on LGBTQIAPN+ issues and may explain the absence or scarcity of research from certain Latin American countries, despite comprehensive search efforts.

In Latin America, TTGD populations face compounded inequalities at the intersection of gender identity, race, class, disability, sexuality, and migration status (170–172). Ethnic and sociocultural diversity coexist with persistent inequities rooted in colonial histories and structural exclusion. Individuals with multiple marginalized identities, such as Indigenous or Afro-descendant heritage, migrant backgrounds, or gender-diverse status, experience systemic barriers, including discrimination, poverty, and invisibility in political and statistical systems (170, 171). Intersectional discrimination is not merely additive but arises from the complex interaction of social hierarchies, and addressing these inequities requires acknowledging that TTGD individuals with additional marginalized identities face distinct obstacles limiting access to resources and opportunities (172).

Compared with global literature, Latin American findings show both convergences and region-specific challenges. Consistent with Scheim et al.'s (3) findings on research gaps regarding transmasculine populations in low- and middle-income countries and limited attention to gender-affirming and general health beyond HIV, this review found a predominance of studies on transfeminine experiences. Holland et al. (163) reported that discrimination and insufficient provider knowledge remain key barriers in PHC globally, mirroring patterns in Latin America. Kearns et al. (4) highlighted that Trans and non-binary youth worldwide navigate complex, costly, and emotionally challenging pathways to gender-affirming care, shaped by family tensions and inconsistent professional support, similar to the dynamics observed in our review. The most peer-based interventions focus narrowly on HIV prevention rather than broader wellbeing or gender affirmation, reflecting a trend in Latin America's limited holistic, community-led initiatives (150). These parallels indicate that the barriers identified in this review reflect broader global inequities in TTGD healthcare, rooted in cisnormativity and structural stigma, yet further shaped in Latin America by colonial legacies and local political, cultural, and health system dynamics that both hinder and sometimes foster more inclusive care.

### 4.5 Health needs and care pathways

Based on the evidence mapped in this scoping review, several priority areas recur in the literature related to structural barriers rooted in cis-heteronormative and biomedical paradigms: Strengthening workforce qualifications to deliver culturally competent, gender-affirming, and humanized care while addressing stigma, discrimination, and knowledge gaps is essential; improving data collection on gender identity, service use, and health outcomes can inform inclusive policies; service organization should reduce regional disparities, decentralize care, and integrate community-led, rights-based approaches, particularly in primary healthcare; expanding access beyond body modifications and enhancing financial protection are critical steps toward structural transformation that recognizes TTGD people as active agents in equitable and sustainable health systems.

### 4.6 Strengths and limitations

Variations in terminology and database indexing may have influenced the scope of the literature retrieved, potentially limiting the comprehensiveness of the review. The inclusion of studies primarily published in English, Spanish, and Portuguese introduces possible language bias, and the search, conducted in 2024, captured only studies published up to that year. The large volume of studies identified, combined with rigorous selection, charting, and analysis procedures, contributed to a lengthy manuscript preparation process.

The predominance of studies from Brazil underscores regional imbalances and highlights the need for broader representation across Latin America. To maintain a clear focus on primary research, non-academic sources, editorials, and commentaries were excluded, although they often provide important contextual insights into barriers to healthcare access. Additionally, as appropriate for scoping reviews and in line with our objective, no formal quality appraisal was conducted; therefore, findings should be interpreted as indicative rather than conclusive.

Despite these limitations, this review provides a comprehensive overview of existing evidence and highlights key priorities for future research and policy to advance equitable healthcare for TTGD populations.

## 5 Conclusion

This scoping review provides the first comprehensive overview of existing evidence on healthcare access for Trans populations in Latin America. The findings highlight persistent structural, economic, and institutional barriers that hinder equitable access to care. Key challenges include fragmented PHC systems, cis-heteronormative and pathologizing approaches, financial and geographic obstacles, and a widespread lack of professional training. These conditions often lead individuals to seek care in emergency settings or engage in risky self-managed health practices, with trust in health systems further compromised by stigma and discrimination.

Promising pathways identified in the literature involve affirming care models with queer-identified providers, community participation, and collaborative planning. Across the studies reviewed, three main directions emerge: (1) comprehensive, gender-affirming care across multiple levels of healthcare, focusing on PHC; (2) intersectional and interprofessional qualification programs, often community-driven, to enhance provider competencies and reduce bias; and (3) the collection and monitoring of regional data to better understand the health needs of diverse TTGD populations.

Future research should address remaining gaps, including longitudinal studies tracking health trajectories, comparative analyses across countries and subregions, and focused investigations on transmasculine and gender-diverse populations. Advancing equitable healthcare in Latin America requires structural transformation that challenges colonial, binary, and biomedical paradigms, centers TTGD voices, and positions these communities as active agents in shaping health systems. Such approaches have the potential to generate innovative, sustainable, and rights-oriented improvements in health equity across the region.
