# Supplementary material for: Access to healthcare for Trans, Travestis, and Gender-Diverse people in Latin America: a scoping review
Source: Front Public Health. 2026 Jan 19;13:1710952. doi: 10.3389/fpubh.2025.1710952 (PMC12833562; doi:10.3389/fpubh.2025.1710952)
Supplement: Supplementary file 1 [file Data_Sheet_1.docx]

***Supplementary Material***

# Search Strategy

The database searches were conducted between May 5 and 9, 2024. The search strategies employed were:

**SCOPUS**: ( TITLE-ABS-KEY ( "Healthcare Quality, Access, and Evaluation" OR accessibility* OR "Accessibilities, Health Services" OR "Access to Health Services" OR "Access to Care" OR "Access to Cares" OR "Care, Access to" OR "Cares, Access to" OR "Accessibility of Health Services" OR "Accessibility, Health Services" OR "Availability of Health Services" OR "Health Services Availability" OR "Access To Care, Health" OR "Access to Health Care" OR "Health Services Geographic Accessibility" OR "Access to Therapy" OR "Access to Therapies" OR "Therapy, Access to" OR "Access to Treatment" OR "Access to Treatments" OR "Treatment, Access to" OR "Access to Medicines" OR "Access to Medicine" OR "Medicine, Access to" OR "Medicines, Access to" OR "Access to Medications" OR "Access to Medication" OR "Medication, Access to" OR "Medication Access" OR "Access, Medication" OR "Medication Accesses" OR "Program Accessibility" OR "Accessibility, Program" OR "Health Inequities" ) ) AND ( TITLE-ABS-KEY ( "trans people" OR "trans woman" OR "trans man" OR travesti OR transgender* OR "Transfeminine" OR "Trans-Feminine Persons" OR "Person, Trans-Feminine" OR "Trans Feminine Persons" OR "Trans-Feminine Person" OR transmasculine OR "Trans-Masculine Persons" OR "Person, Trans-Masculine" OR "Trans Masculine Persons" OR "Trans-Masculine Person" OR "Two-Spirit Persons" OR "Person, Two-Spirit" OR "Two Spirit Persons" OR "Two-Spirit Person" OR transsexual* OR "Gender Fluidity" OR "Fluidity, Gender" OR "Gender Fluidities" OR "Travesti" ) ) AND ( TITLE-ABS-KEY ( "Latin America" OR "South America" OR "Central America" OR "Belize" OR "Costa Rica" OR "El Salvador" OR "Guatemala" OR "Honduras" OR "Mexico" OR "Nicaragua" OR "Panama" OR "Argentina" OR "Bolivia" OR "Brazil" OR "Chile" OR "Colombia" OR "Ecuador" OR "French Guiana" OR "Guyana" OR "Paraguay" OR "Peru" OR "Suriname" OR "Uruguay" OR "Venezuela" OR "Cuba" OR "Dominican Republic" OR "Haiti" OR "Guadeloupe" OR "Martinique" OR "Puerto Rico" OR "Saint-Barthélemy" OR "Saint-Martin" ) )

**WEB OF SCIENCE:** "trans people" OR "trans woman" OR "trans man" OR travesti OR Transgender* OR “Transfeminine” OR "Trans-Feminine Persons" OR "Person, Trans-Feminine" OR "Trans Feminine Persons" OR "Trans-Feminine Person" OR Transmasculine OR "Trans-Masculine Persons" OR "Person, Trans-Masculine" OR "Trans Masculine Persons" OR "Trans-Masculine Person" OR "Two-Spirit Persons" OR "Person, Two-Spirit" OR "Two Spirit Persons" OR "Two-Spirit Person" OR Transsexual* OR "Gender Fluidity" OR "Fluidity, Gender" OR "Gender Fluidities" OR "Travesti" AND “Healthcare Quality, Access, and Evaluation” OR Accessibility* OR “Accessibilities, Health Services” OR “Access to Health Services” OR “Access to Care” OR “Access to Cares” OR “Care, Access to” OR “Cares, Access to” OR “Accessibility of Health Services” OR “Accessibility, Health Services” OR “Availability of Health Services” OR “Health Services Availability” OR “Access To Care, Health” OR “Access to Health Care” OR “Health Services Geographic Accessibility” OR “Access to Therapy” OR “Access to Therapies” OR “Therapy, Access to” OR “Access to Treatment” OR “Access to Treatments” OR “Treatment, Access to” OR “Access to Medicines” OR “Access to Medicine” OR “Medicine, Access to” OR “Medicines, Access to” OR “Access to Medications” OR “Access to Medication” OR “Medication, Access to” OR “Medication Access” OR “Access, Medication” OR “Medication Accesses” OR “Program Accessibility” OR “Accessibility, Program” OR “Health Inequities” AND "Latin America" OR “South America” OR “Central America” OR “Belize” OR “Costa Rica” OR “El Salvador” OR “Guatemala” OR “Honduras” OR “Mexico” OR “Nicaragua” OR “Panama” OR “Argentina” OR “Bolivia” OR “Brazil” OR “Chile” OR “Colombia” OR “Ecuador” OR “French Guiana” OR “Guyana” OR “Paraguay” OR “Peru” OR “Suriname” OR “Uruguay” OR “Venezuela” OR “Cuba” OR “Dominican Republic” OR “Haiti” OR “Guadeloupe” OR “Martinique” OR “Puerto Rico” OR “Saint-Barthélemy” OR “Saint-Martin”

**PUBMED**: (((accessibility[Title/Abstract]) OR ("Health Care Quality, Access, and Evaluation" [MeSH Terms] OR "Health Services Accessibility" [MeSH Terms] OR "Health Inequities" [MeSH Terms]))) AND (("transgender" [TIAB] OR "trans people" [TIAB] OR "trans woman" [TIAB] OR "trans man" [TIAB] "travesti" [TIAB] OR transsexual [TIAB]) OR ("Transgender Persons" [MeSH Terms] OR "Health Services for Transgender Persons" [MeSH Terms] OR "Transsexualism" [MeSH Terms])) AND (((("Latin America"[Mesh]) OR "Caribbean Region"[Mesh]) OR "Central America"[Mesh]) OR "South America"[Mesh])

The search strategy used in the SciELO and LILACS databases included search strings in Portuguese, English, and Spanish, following the specific guidelines of each database.

**LILACS**: ((mh:((personas transgénero) OR (minorías sexuales y de género) OR (servicios de salud para las personas transgénero) OR (transexualidad) OR (travestismo)) OR (transgénero) OR (personas trans) OR (travesti) OR (transexual)) AND (mh:((accesibilidad a los servicios de salud) OR (calidad, acceso y evaluación de la atención de salud) OR (equidad en salud)) OR ((acceso) OR (accesibilidad)))) OR ((mh:((transgender persons) OR (sexual AND gender minorities) OR (health services for transgender persons) OR (transsexualism) OR (transvestism)) OR (transgender) OR (trans people) OR (travesti) OR (transsexual)) AND (mh:((health services accessibility) OR (health care quality, access, AND evaluation) OR (health equity)) OR ((access) OR (accessibility)))) OR ((mh:((pessoas transgênero) OR (minorias sexuais e de gênero) OR (serviços de saúde para pessoas transgênero) OR (transexualidade) OR (travestilidade)) OR (transgênero) OR (pessoas trans) OR (travesti) OR (transsexual)) AND (mh:((acessibilidade aos serviços de saúde) OR (qualidade, acesso e avaliação da assistência à saúde) OR (equidade em saúde)) OR ((acesso) OR (acessibilidade)) )) AND ( db:("LILACS"))

**SCIELO**: ((kw:((Personas Transgénero) OR (Minorías Sexuales y de Género) OR (Servicios de Salud para las Personas Transgénero) OR (Transexualidad) OR (Travestismo)) OR (Transgénero) OR (personas trans) OR (travesti) OR (transexual)) AND (kw:((Accesibilidad a los Servicios de Salud) OR (Calidad, Acceso y Evaluación de la Atención de Salud) OR (Equidad en Salud)) OR ((acceso) OR (accesibilidad)))) OR ((kw:((Transgender Persons) OR (Sexual and Gender Minorities) OR (Health Services for Transgender Persons) OR (Transsexualism) OR (Transvestism)) OR (transgender) OR (trans people) OR (travesti) OR (transsexual)) AND (kw:((Health Services Accessibility) OR (Health Care Quality, Access, and Evaluation) OR (Health Equity)) OR ((access) OR (accessibility)))) OR ((kw:((pessoas transgênero) OR (minorias sexuais e de gênero) OR (serviços de saúde para pessoas transgênero) OR (transexualidade) OR (travestilidade)) OR (transgênero) OR (pessoas trans) OR (travesti) OR (transsexual)) AND (kw:((acessibilidade aos serviços de saúde) OR (qualidade, acesso e avaliação da assistência à saúde) OR (equidade em saúde)) OR ((acesso) OR (acessibilidade)) ))

# Theoretical Framework Rationale

| **RATIONALE** | | | | |
| --- | --- | --- | --- | --- |
| **Enabling/ Disabling Factors** | **Availability/ Accessibility** | **Healthcare users’ and providers' perspectives** | Representation of the presence or absence of a health service or good at the appropriate location and at the time it is needed. This dimension broadly encompasses the geographic relationship between physical health institutions and the individual requiring care, such as distance and transportation options. The transportation resources available to health professionals, as well as their willingness to travel to care for individuals in need, can also be considered in this analysis (Sanchez & Ciconelli, 2012). This is synonymous with the concepts of accessibility and availability as described by Soares (2013). | Example indicators: type of service used (hospital-based, medical, dental, emergency, home care), location where care was provided (residence, office, clinic, hospital), purpose of care (preventive, curative), % of at-risk population who did or did not visit a physician within a given timeframe, number of beds, equipment. Write the results mentioned and related to the experiences of health professionals separately from those mentioned by users, each in its own category. |
|  | **Payment capacity/ Financing** | **Healthcare users’ and providers' perspectives** | Relationship between the cost of services and the user's ability to pay for them, or the assurance of service provision (Soares, 2013); relationship between the cost of using health services and individuals’ ability to pay (Sanchez, Ciconelli, 2012). | Example indicators: income, sources of income, health insurance coverage, characteristics of the regular source of care, and direct and indirect health-related costs. Write the results mentioned and related to the experiences of health professionals separately from those mentioned by users, each in its own category. |
|  | **Information** | **Healthcare users’ and providers' perspectives** | Degree of asymmetry between the knowledge of the patient and that of the health professional (Sanchez & Ciconelli, 2012). Described exclusively by Sanchez & Ciconelli. | Example indicators: educational level, knowledge, and sources of information. Write the results mentioned and related to the experiences of health professionals separately from those mentioned by users, each in its own category. |
|  | **Acceptability** | **Healthcare users’ and providers' perspectives** | Relationship between users’ attitudes toward the personal characteristics, trustworthiness, and expected practices of service providers, and providers’ attitudes regarding acceptable personal characteristics and behaviors of users (Soares, 2013). | Example indicators: values and attitudes toward the organization of the service, healthcare professionals, care delivery processes, trust in providers, and health-related expectations or anxiety. Write the results mentioned and related to the experiences of health professionals separately from those mentioned by users, each in its own category. |
|  | **Adequacy** | **Healthcare users’ perspectives** | Relationship between how resources are organized to receive users (e.g., scheduling systems, care protocols) and the user’s ability to adapt to these factors, as well as their perception of appropriateness (Soares, 2013). Described exclusively by Soares. | Example indicators: waiting time for surgery, meeting or not meeting eligibility criteria for accessing a service (e.g., surgery), among others. |
| **Health needs** | **Normative** | | It is regulated by the state and/or health professionals; what the user feels is necessary does not necessarily converge with what the system establishes as a need. | A national health guideline recommends that all adults over 50 undergo colorectal cancer screening. A 55-year-old person feels healthy and sees no reason to get tested, but from a public health perspective, screening is a normative need due to the risk associated with their age group. |
|  | **Felt** | | Perceiving the need for a service or good based on one’s health perception (user). | An individual begins to experience persistent abdominal discomfort and bloating. Although they have not consulted a health professional or received a diagnosis, they believe they may need medical attention or dietary changes. This personal recognition of a possible health issue, independent of formal assessment, represents a felt need. |
|  | **Expressed** | | Demand for services by individuals, corresponding to the felt need, is transformed into action. Feeling the need does not equate to experiencing the problem. One may feel the problem (e.g., esophagitis/gastric ulcer) and believe that herbal tea X is the necessary remedy, or may choose not to use proton pump inhibitors (e.g., rumors about Omeprazole causing cancer). | Following the persistent abdominal discomfort and bloating, the individual decides to schedule a medical appointment and requests diagnostic tests to investigate the symptoms. By seeking professional help, they have transformed their felt need into an expressed need through concrete action. |
|  | **Comparative** | | Needs that are not met when compared (felt/normative/expressed). | Two groups have similar perceived (felt) and recommended (normative) mental health needs. In one group, services are available and accessed (expressed need met), while in the other, services are lacking and needs go unmet. This difference illustrates a comparative need, unmet needs in one group compared to another, despite similar demands. |
| **Individual Health Behaviors** | **Healthcare users’ and providers’ experiences** | | The use of health services can be viewed as a type of individual health behavior. (...) Health behaviors encompass self-medication, adherence to treatment, and the utilization of care services that involve medications and other interventions (Soares, 2013), such as avoiding seeking health services and/or opting for alternative pathways. | What determines behaviors is the perspective of each user (beliefs, cultural aspects, principles, and values). Write the results mentioned and related to the experiences of health professionals separately from those mentioned by users, each in its own category. |

**REFERENCES**

1. Andersen RM & Davidson PL. Improving access to care in America: individual and contextual indicators. 2007.
2. Sanchez RM & Ciconelli RM. Concepts of access to health care [in Portuguese]. Rev Panam Salud Publica. 2012;31(3):260–8.
3. Soares L. Access to dispensing services and medicines: theoretical model and empirical elements [PhD thesis, in Portuguese]. Florianópolis: Federal University of Santa Catarina; 2013. 249 p
